# Multi‐Mode and Dynamic Persistent Luminescence from Metal Cytosine Halides through Balancing Excited‐State Proton Transfer

**DOI:** 10.1002/advs.202200992

**Published:** 2022-04-10

**Authors:** Guowei Xiao, Xiaoyu Fang, Yu‐Juan Ma, Dongpeng Yan

**Affiliations:** ^1^ Beijing Key Laboratory of Energy Conversion and Storage Materials College of Chemistry and Key Laboratory of Radiopharmaceuticals, Ministry of Education Beijing Normal University Beijing 100875 P. R. China

**Keywords:** metal halides, persistent luminescence, room‐temperature phosphorescence, smart materials, thermally activated delayed fluorescence

## Abstract

Persistent luminescence has attracted great attention due to the unique applications in molecular imaging, photodynamic therapy, and information storage, among many others. However, tuning the dynamic persistent luminescence through molecular design and materials engineering remains a challenge. In this work, the first example of excitation‐dependent persistent luminescence in a reverse mode for smart optical materials through tailoring the excited‐state proton transfer process of metal cytosine halide hybrids is reported. This approach enables ultralong phosphorescence and thermally activated delayed fluorescence emission colors highly tuned by modulation of excitation wavelength, time evolution, and temperature, which realize multi‐mode dynamic color adjustment from green to blue or cyan to yellow‐green. At the single crystal level, the 2D excitation/space/time‐resolved optical waveguides with triple color conversion have been constructed on the organic‐metal halide microsheets, which represent a new strategy for multi‐dimensional information encryption and optical logic gate applications.

## Graphical abstract

Reversed excitation‐dependent persistent luminescence can be obtained in metal cytosine halides by controlling the process of excited‐state proton transfer (ESIPT). The metal cytosine halides further exhibit multi‐mode (excitation/space/time) triple‐color luminescent conversion, which have promising applications in multi‐dimensional information encryption and photonic logic gates.

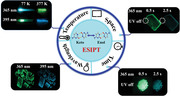

## 1 Introduction

Molecular persistent luminescence, particularly for room‐temperature phosphorescence (RTP) and thermally activated delayed fluorescence (TADF), has attracted great attention due to its unique applications in sensors,^[^ ^1^, ^2^, ^3^ ^]^ molecular imaging,^[^ ^4^, ^5^ ^]^ photodynamic therapy,^[^ ^6^ ^]^ information storage,^[^ ^7^, ^8^ ^]^ and security encryption.^[^ ^9^, ^10^, ^11^ ^]^ Owing to the combined effects of the spin‐forbidden transition of the intersystem crossing (ISC) process and the susceptibility of the triplet excitons to molecular motion and vibration, obtaining highly efficient and ultralong RTP emission has been challenging historically. Recent years have witnessed efforts to boost ultralong RTP and stabilize the triplet excitons via molecular design and assembly methods, including crystal engineering,^[^ ^12^, ^13^, ^14^, ^15^ ^]^ H‐aggregates,^[^ ^16^, ^17^, ^18^ ^]^ host‐guest doping,^[^ ^19^, ^20^, ^21^ ^]^ constructions of the polymer matrix,^[^ ^22^, ^23^, ^24^ ^]^ metal halides,^[^ ^25^, ^26^, ^27^, ^28^ ^]^ and metal‐organic frameworks.^[^ ^29^, ^30^ ^]^ To date, most persistently luminescent materials usually produce only a single emission color, limiting opportunities to tune a wide range of colors via multi‐mode stimuli. Only few systems with individual temperature‐^[^ ^31^ ^]^ and time‐dependent^[^ ^32^ ^]^ emission have been reported. For example, Yang et al. reported an organic crystal whose changeable afterglow color featured well separated TADF and RTP.^[^ ^33^ ^]^ As another fashion, the excitation‐dependent photoemission has also aroused much interest since it can facilitate the complex goals of multi‐channel imaging.^[^ ^34^ ^]^ Yuan's group developed the excitation‐dependent afterglow by forming diversified RTP emissive species with different decay lifetimes.^[^ ^35^ ^]^ Generally, the excitation‐dependent photoemission undergoes a red‐shift upon increase of excitation wavelength, a positive‐forward behavior which is reasonable given the energy matching between the excitation and emission bands, and which has also been observed widely in luminescent carbon dots^[^ ^36^ ^]^ and coordination polymers.^[^ ^37^ ^]^ To meet the increasing demand to develop high‐level security protection such as anti‐counterfeiting encryption, we consider whether the emission can exhibit the converse behavior (blue‐shifted emission with increasing excitation) for new type of multicolor luminescence. This characteristic would enable significant enrichment of optical information modes, but is still rather rare among photoluminescent materials, particularly those featuring persistent luminescence (Table S1, Supporting Information).

As a classical photochemical mode in the biological process, the excited‐state intramolecular and/or intermolecular proton transfer (ESIPT) can be commonly found in nature.^[^ ^38^, ^39^, ^40^ ^]^ The classical ESIPT, that is induced by ultraviolet or visible light, involves a tautomeric transformation from excited keto‐form to enol‐form, which elicits a photoemission that is red‐shifted relative to the excitation (Figure S1, Supporting Information). This brings about the unique four‐level energy state cycle and makes possible the realization of multiple and transformable emissions.^[^ ^41^ ^]^ During last few years, research on its photophysical properties and fundamental proton transfer dynamics is well documented, contributing to the enriched ESIPT molecular families in both solution and solid states.^[^ ^42^ ^]^ In turn, these advancements have enabled the development of new applications as components in solar concentrators,^[^ ^43^ ^]^ optical memory,^[^ ^44^ ^]^ dual emitters,^[^ ^45^ ^]^ and green fluorescent proteins.^[^ ^46^ ^]^

Cytosine, a well‐known biological DNA base containing both –NH and ═O units, presents an avenue by which the excited state energy level may be adjusted via ESIPT. While the keto, enol, and even keto‐imino tautomers of cytosine have been well characterized in solution and gas phases via experiments and theoretical calculations,^[^ ^47^ ^]^ the solid‐state photophysical properties remain less understood. In principle, the cytosine has the potential to present RTP properties, due to the *n*–π* transition and strong intermolecular interactions.^[^ ^48^ ^]^ Moreover, the prevalence of intermolecular interaction sites and the diversity of structural tautomerism endow the cytosine molecule with great opportunity for assembly into the hybrid materials. In order to leverage the respective advantages of both ESIPT and RTP, we have aimed to construct new types of cytosine‐based self‐assembled systems with metal halides which enable divisible tunability of persistent luminescence.

The last few decades have witnessed a marked expansion of organic‐metal halides for optoelectronic materials applications, broadly exemplified in solar cells and light‐emitting devices.^[^ ^49^, ^50^ ^]^ Additionally, the introduction of heavy metals and halides units into molecular phosphors has facilitated new avenues for tailoring the spin–orbit coupling (SOC) and achieving multicolor luminescence effectively.^[^ ^25^, ^51^ ^]^ Herein, we selected a series of metal halides (Cd/ZnX_2_ (X = Cl, Br)) to assemble with cytosine, and obtained a range of organic‐metal halide hybrids, namely Cy‐CdCl_2_, Cy‐CdBr_2_, Cy‐ZnCl_2_, and Cy‐ZnBr_2_ respectively. Interestingly, the organic‐metal halides present a rather unusual backward excitation‐dependent persistent luminescence spanning a wide emission range from blue to yellow‐green based on the potential ESIPT. The afterglow, which is dependent on multiple modes (excitation, temperature, and time), further renders the well‐organized 2D organic‐metal microstructures to be highly tunable optical waveguides capable of logic manipulation at the single crystal level. To the best of our knowledge, this work serves as the first example of multiple‐stimuli‐responsive and colorful persistent luminescence based on the facile modulation of ESIPT in organic‐metal halides.

## 2 Results and Discussion

### 2.1 Synthesis and Crystal Structure of Organic‐Metal Halide

**Figure 1:**
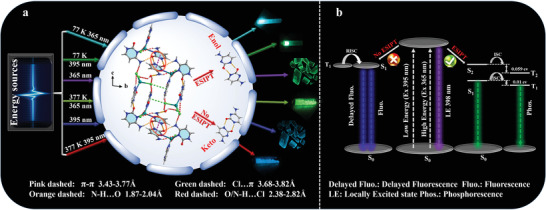
Schematic representation for the multi‐mode persistent luminescence of Cy‐CdCl_2_ under different energy. a) Multi‐mode dynamic persistent luminescence of Cy‐CdCl_2_ organic‐metal hybrid. b) The proposed mechanism of hybrid material Cy‐CdCl_2_ for multicolor ultralong phosphorescence, which is tuned by excitation wavelengths.

Assembly of CdCl_2_ and cytosine (molar ratio, 1: 2) in water solution (10 mL) was carried out at room temperature to afford bulk microcrystals (designated Cy‐CdCl_2_). The single‐crystal X‐ray diffraction (SCXRD) data reveal that Cy‐CdCl_2_ crystallizes in the triclinic with *P*ī space group. The asymmetric unit contains two Cd^2+^, four Cl^–^, four coordinated cytosine molecules, and a lattice water molecule. Both Cd centers are located in a tetrahedral coordination environment with [CdN_3_Cl] for both Cd1 and Cd2. All N atoms are contributed by cytosine cations, and decorated on Cd^2+^ as terminal ligands forming two crystallographically independent units, [(C_4_H_5_N_3_O)_3_CdCl]^+^ and [(C_4_H_5_N_3_O)CdCl_3_]^–^, respectively. The co‐crystallized organic metal halide is organized mainly via *π*–*π* stacking, Cl···*π* and H‐bonding interactions. The *π*–*π* stacking between adjacent cytosine molecules is within 3.434–3.773 Å, while the Cl···*π* distances range from 3.684 to 3.815 Å. The H‐bonding interactions include both N/O–H···Cl (2.30–2.82 Å) and N–H···O (1.87–2.04 Å). The C═O distance ranges from 1.228 to 1.242 Å, confirming that all cytosine in Cy‐CdCl_2_ exhibit the keto form in the ground state.^[^ ^52^ ^]^ Thus, the cytosine molecules are fixed in a rigid metal halide environment via strong coordination bonds and intermolecular interactions, which may effectively suppress routes for non‐radiative decay (**Figure** **1**a).

FT‐IR spectra (Figure S2a,c, Supporting Information) present sharp peaks at 1670/1630 cm^–1^ and 1721/1640 cm^–1^, attributable to the C═O in cytosine and consistent with a predominant ketone‐form manifesting as in Cy‐CdCl_2_ under ambient conditions. UV–vis absorption spectra show (Figure S2b,d, Supporting Information) that the sub‐300 nm absorption peak of the *n*–π* transition is attributable to the carbonyl group, while the wide band from 370 to 410 nm indicates mainly the *π*–π* transition of the keto form.^[^ ^52^, ^53^, ^54^, ^55^ ^]^ Powder X‐ray diffraction (PXRD; Figure S3b, Supporting Information) demonstrates that the product is a single‐phase Cy‐CdCl_2_ sample.

### 2.2 Reversed Excitation‐Dependent Persistent Luminescence and ESIPT Mechanism

**Figure 2:**
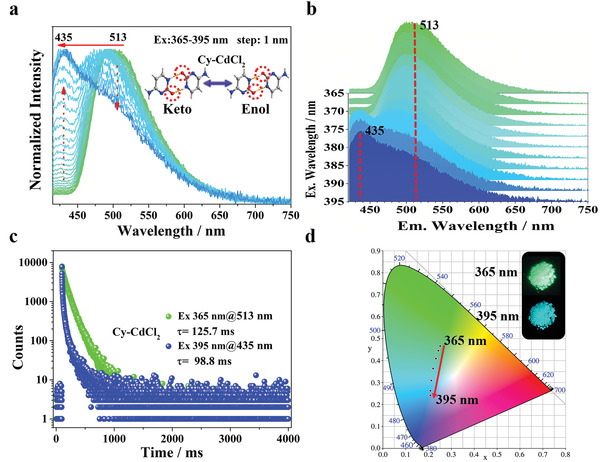
Irradiation‐responsive persistent luminescence of Cy‐CdCl_2_ with different wavelengths. a) Excitation‐phosphorescence mapping of Cy‐CdCl_2_ under ambient conditions. b) The RTP spectra of the Cy‐CdCl_2_ under the excitation at 365 nm (green) and 395 nm (blue), respectively. c) Decay curves of Cy‐CdCl_2_ at 435 and 513 nm. d) CIE coordinate diagram of Cy‐CdCl_2_ by changing the excitation wavelengths.

Excitation by 365 nm UV light causes the solid‐state Cy‐CdCl_2_ to emit bright blue photoluminescence (PL) at room temperature. The photophysical properties were studied via PL spectroscopy conducted under both prompt and delayed (acquired after 1 ms of excitation) modes. Interestingly, irradiation by different wavelengths of UV light evidences that the photoemission of the metal halide hybrid is highly tunable under ambient conditions. Excitation by 365 nm UV light produces bright UV/blue fluorescence and green persistent luminescence (Figure S4, Supporting Information). In the prompt PL spectra, the Cy‐CdCl_2_ exhibits a colorful fluorescence in the blue, green and red regions (Figure S5, Supporting Information), and increasing the excitation wavelength (from 260 to 540 nm) accordingly elicits a red‐shifting of the emission peak from 330 to 616 nm: the positive‐forward excitation‐dependent fluorescence indicates that the metal halide possesses multiple energy levels.^[^ ^56^ ^]^ However, the delayed PL mapping spectra (**Figure** **2**a,b) indicates that increasing the excitation wavelength from 365 to 395 nm induces a gradual decrease in the maximum afterglow peak at 513 nm, which is accompanied by a progressive increase in the emission at 435 nm. This finding can be validated by quantification of the spectra using the Commission Internationale de l'Eclairage (CIE) coordinates, which indicate that the afterglow color changes from green to blue, consistent with that observed by the naked eye (Figure 2d). In stark contrast to state‐of‐the‐art persistent luminescent materials which typically exhibit positive‐forward excitation‐dependent RTP (Table S1, Supporting Information), our findings in this study represent a unique luminescent property. To the best of our knowledge, prior to this work, there has never been a report on afterglow color of a material shifting from long to short wavelengths with increasing excitation wavelength.

**Figure 3:**
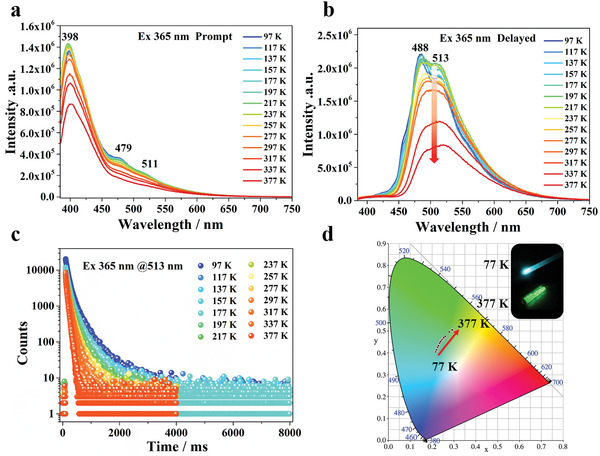
Irradiation‐responsive RTP of Cy‐CdCl_2_ with different temperatures upon 365 nm lamp. a) The PL spectra of Cy‐CdCl_2_ at different temperatures ranging from 97 to 377 K under the prompt mode. b) The PL spectra of Cy‐CdCl_2_ at different temperatures ranging from 97 to 377 K under the delayed mode. c) The time‐resolved decay profiles at 513 nm from 97 to 377 K. d) CIE coordinate diagram of Cy‐CdCl_2_ under excitation by a 365 nm UV lamp at different temperatures ranging from 97 to 377 K.

To better understand the mechanism of the reversed excitation‐dependent persistent luminescence above, we have collected and analyzed detailed prompt and delayed spectra at different temperatures. In the prompt mode (**Figure** **3**a), upon excitation at 365 nm, the emission bands are mainly located at 398, 479, and 511 nm, which correspond to singlet excitons of both keto (short wavelength) and enol (long wavelength) forms, respectively. Temperature‐dependent spectra do not give evidence for any marked shift of the steady‐state prompt emission, except for a slight increase in the intensities at 479 and 511 nm. Persistent luminescence under the delayed mode is mainly concentrated at 488 and 513 nm at 97 K. Upon raising the temperature from 97 to 377 K, the delayed emission undergoes a decrease in intensity and a red‐shift toward 520 nm, reflecting enhanced molecular vibration. The decay curves show the luminescent lifetimes of the emissions at 488 and 513 nm correspond to tens to hundreds of milliseconds (from 490.8 to 10.1 ms and 342.7 to 44.1 ms, respectively). Thus, the persistent luminescence can be conclusively ascribed to the ultralong phosphorescence from triplet excitons (Figure 3b,c).

**Figure 4:**
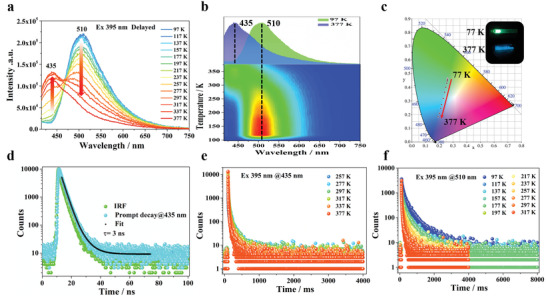
Irradiation‐responsive persistent luminescence of Cy‐CdCl_2_ with different temperatures upon 395 nm lamp. a) The PL spectra of Cy‐CdCl_2_ at different temperatures ranging from 97 to 377 K under the delayed mode. b) The RTP spectra of the Cy‐CdCl_2_ powder at the temperatures of 97 K (blue) and 377 K (red), respectively. c) CIE coordinate diagram of Cy‐CdCl_2_ under excitation by a 395 nm UV lamp at different temperatures spanning 97 to 377 K. d) The nanosecond‐scale prompt lifetime decay profiles of Cy‐CdCl_2_ at 435 nm. e) The time‐resolved decay profiles at 435 nm from 257 to 377 K. f) The time‐resolved decay profiles at 510 nm from 97 to 317 K.

395 nm excitation elicits nearly identical prompt spectra, with a major peak occurring at 435 nm under low, ambient and high temperatures (typically corresponding to 97, 297, and 377 K, Figures S6 and S7a–c, Supporting Information). It is also notable that this peak appears as a shoulder band upon the 365 nm excitation, suggesting some relation to the photoemission from the keto‐form aggregation state. Delayed spectra induced by 395 nm excitation feature an emission band concentrated mainly at 510 nm in the temperature range spanning 97–217 K (**Figure** **4**a,b, Figure S7d, Supporting Information). This emission band strongly resembles that observed by 365 nm excitation. However, upon further increase of the temperature from 217 to 377 K, the emission at 510 nm gradually decreases, while a new peak at 435 nm overlapping with the prompt PL spectra increases (Figures S6e,f and S3c, Supporting Information). Time‐resolved decay spectra tests monitored at 435 nm determine that the lifetimes of the short‐lived and long‐lived excited states under ambient conditions are 3 ns and 98.8 ms, respectively (Figure 4d,e), indicating the coexistence of prompt fluorescence and TADF of Cy‐CdCl_2_.

From a structural perspective, the intermolecular N–H···O hydrogen bond in the cytosine units makes the ESIPT process possible, leading to the keto‐to‐enol structural transformation. Different UV energies can be used to balance the ESIPT process and thus control the ratio of keto‐form to enol‐form, thereby tuning the long‐afterglow colors of the materials under ambient conditions. The high excitation energies (<380 nm) promote the structural transformation from the original keto‐ to the new enol‐isomer; with lower excitation energies (>380 nm), the keto‐form manifests more predominantly in the steady state of cytosine, as observed in the crystal structure. To gain further insights into the unusual photophysical processes, density function theoretical (DFT) calculation was conducted on the keto‐ and enol‐isomers of the cytosine molecule, which serves as the main center for luminescence of Cy‐CdCl_2_. The calculated T_1_→S_0_ energy gap values (≈2.82 eV for keto and ≈2.06 eV for enol) are in good qualitative agreement with corresponding experimental observations (≈2.85 eV for keto and ≈2.42 eV for enol). Therefore, the UV/blue emission at 398 nm can be attributed to the keto‐form locally excited state, and the green emission (511 nm) is related to the enol‐form based on the ESIPT process (Figure 3a).^[^ ^57^ ^]^ The proposed mechanism of the Cy‐CdCl_2_ hybrid for multicolor persistent luminescence based on the reversed excitation dependence is shown in Figure 1b.

### 2.3 Afterglow Properties of Analogous Organic‐Metal Halides

Until now, developing purely organic RTP molecules has been accomplished strictly via manipulation of the strong heavy‐atom effect (HAE), which can greatly enhance SOC and facilitate ISC process.^[^ ^58^, ^59^, ^60^ ^]^ However, because the noble metals‐based HAE substantially accelerates the radiation rate, it is not conducive to obtaining an ultralong lifetime. Therefore, a more effective strategy to achieve long afterglow emission is to implement a medium HAE strategy. The HAE effect exhibited by zinc is weaker than that exhibited by cadmium, leading us to select zinc as a key component for constructing metal halide hybrid materials. Substituting ZnCl_2_, ZnBr_2_, and CdBr_2_ metal salts for CdCl_2_ led to the formation of Cy‐ZnCl_2_, Cy‐ZnBr_2_, and Cy‐CdBr_2_, respectively (Figures S8 and S9, Supporting Information). The assembled structures of these products feature some differences compared to the Cy‐CdCl_2_.

The SCXRD (Figure S8, Tables S2, S3, S5, S6, Supporting Information) data reveal that Cy‐ZnCl_2_ and Cy‐ZnBr_2_ both crystallize in the *C*2/c space group of the orthorhombic system with isostructure. The asymmetric unit contains 1/2 Zn^2+^, two X^–^ (X = Cl, Br), and two cytosine molecules (one of which is protonated). Zn is centered in a [ZnX_4_] tetrahedral coordination environment which forms a mononuclear structure. Bond lengths around the Zn centers span 2.249–2.280 Å for Cy‐ZnCl_2_ and 2.389–2.409 Å for Cy‐ZnBr_2_. The C═O distance ranges from 1.227–1.253 Å, indicating that all cytosine molecules exhibit the keto form in the ground state. The [ZnX_4_]^2–^ unit and free keto cytosine molecules assemble into the 0D metal halide hybrid via *π*–*π* stacking, X···*π* and H‐bonding interactions. The *π*–*π* stacking distances span 3.495–3.799 Å for Cy‐ZnCl_2_ and 3.446–3.825 Å for Cy‐ZnBr_2_. The Cl···*π* distance is 3.632 Å while the Br···*π* distance is 3.649 Å. H‐bonding interactions can be divided into N–H···X interactions (Cy‐ZnCl_2_: 2.52 Å and Cy‐ZnBr_2_: 2.59 Å) and N–H···O/N interactions (Cy‐ZnCl_2_: 1.79–1.92 Å; Cy‐ZnBr_2_: 1.92–2.12 Å).

Cy‐CdBr_2_ crystallizes (Figure S9c,d, and Table S8, Supporting Information) in the triclinic structure with a *P*ī space group. The asymmetric unit consists of one Cd^2+^, two Br^–^, and two cytosine molecules. Cd is centered in [CdN_2_Br_2_] and exhibits tetrahedral geometry with Cd‐Br lengths of 2.582–2.592 Å and Cd‐N lengths of 2.243–2.281 Å. The C═O distances range from 1.230–1.239 Å, indicating that both cytosine molecules exhibit the keto form. Such units form a mononuclear structure, which further assemble into a 1D supramolecular structure via *π*–*π* stacking, Br···*π* and H‐bonding interactions. The *π*–*π* stacking distances are in the range of 3.477–4.053 Å while the Br···*π* distance is 3.991 Å. H‐bonding interactions can be divided into N–H···Br interactions spanning 2.68–2.90 Å and N–H···O interactions spanning 1.91–2.01 Å.

**Figure 5:**
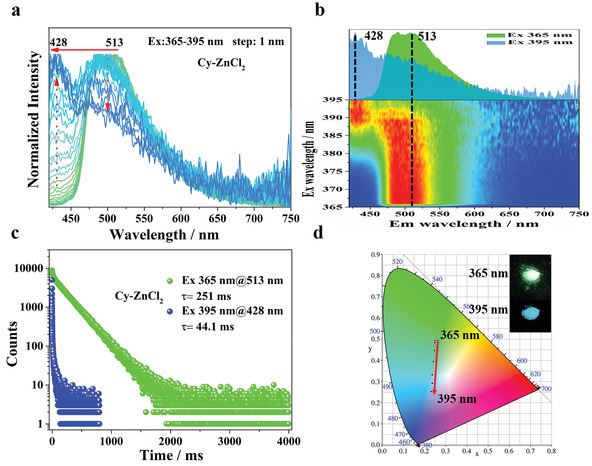
Irradiation‐responsive persistent luminescence of Cy‐ZnCl_2_ with different wavelengths. a) The RTP spectra of the Cy‐ZnCl_2_ under the excitation at 365 nm (green) and 395 nm (blue), respectively. b) Excitation‐phosphorescence mapping of the Cy‐ZnCl_2_ under ambient conditions. c) Decay curves of Cy‐ZnCl_2_ at 428 and 513 nm. d) CIE coordinate diagram of Cy‐ZnCl_2_ as a function of the excitation wavelengths.

The reversed excitation wavelength‐dependent properties observed in the as‐prepared Cy‐ZnCl_2_, Cy‐ZnBr_2_, and Cy‐CdBr_2_ are similar to those in the Cy‐CdCl_2_, although the tuning ranges of luminescence are different [513–428 nm for Cy‐ZnCl_2_ (**Figure** **5**), 505–476 nm (Figure S10c, Supporting Information) for Cy‐ZnBr_2_, and 527–490 nm for Cy‐CdBr_2_ (Figure S11c, Supporting Information)]. Meanwhile, the HAE of the Br atom accelerates the radiative rate for Cy‐ZnBr_2_ and Cy‐CdBr_2_, substantially reducing their phosphorescence lifetimes to 37.3@476 nm (or 38.3 ms@505 nm) (Figure S12a, Supporting Information) and 18.9@490 nm (or 18.6 ms@527 nm) (Figure S12b, Supporting Information), respectively.

**Figure 6:**
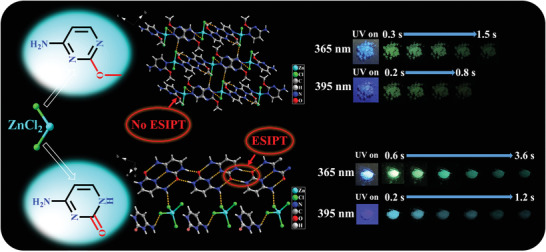
Single‐crystal structures of the two target compounds and afterglow photographs. The packing modes and afterglow photographs for crystalline samples of Me‐Cy‐ZnCl_2_ (upper) and Cy‐ZnCl_2_ (lower), where the occurrence of ESIPT has been circled.

To further detect the role of ESIPT in inverting the excitation wavelength dependence of persistent luminescence, we selected the molecule 2‐methoxy‐4‐pyrimidinamine (Me‐Cy), in which one methyl is grafted to a C═O group for a comparative examination. The methylated metal halide (Me‐Cy‐ZnCl_2_) was synthesized similar to that of Cy‐ZnCl_2_ (**Figure** **6**), in a manner wherein potential proton transfer is absent in the crystal structure (Tables S2, S4, S9, Supporting Information). Upon excitation in the range of 330–410 nm, Me‐Cy‐ZnCl_2_ displays a major emissive band at 525 nm attributed to the delayed PL spectra, but does not show the inversed excitation wavelength dependence (Figure S13, Supporting Information). Moreover, An and Huang's group has reported the RTP properties of the pristine cytosine ligands,^[^ ^47^ ^]^ which consistently present only positively‐forward excitation‐dependent afterglow emission. X‐ray diffraction analysis of single crystal cytosine indicates that the neutral keto molecules in the same plane are strictly restricted through multiple intermolecular interactions. We propose that the ionic or protonated cytosine molecules are necessary to the ESIPT process, which can be accelerated by a highly polarized environment. Thus, the pristine cytosine solid cannot achieve the reversed excitation‐tunable color characteristics.

To better understand the electronic structures and multi‐color emissive mechanism of the Cy‐Cd/ZnX_2_ organic‐metal halides, we obtained their density of states (DOS) distributions by using DFT calculations (Figures S14–S16, Supporting Information). Taking Cy‐CdCl_2_ as an example, frontier orbital analyses show that the highest occupied molecular orbital (HOMO) and the lowest unoccupied molecular orbital (LUMO) together with HOMO‐1 and LUMO+1 are predominantly localized on the C/O/N *2p* atomic orbitals of the organic cytosine, suggesting that both the *π*–π* and *n*–π* transitions are contributing sources to the fluorescence and phosphoresce of Cy‐CdCl_2_. Such multi‐channel transition fashions are consistent with our experimentally observed excitation wavelength dependence. In Cy‐CdBr_2_, the inorganic [CdN_2_Br_2_] tetrahedron also participates in the HOMO and LUMO (Figure S15, Supporting Information); near the Fermi level, the partial DOS is mainly attributable to both the Br and cytosine, which indicates that the HAE of halide units is sufficient to affect the photophysical properties of Cy‐CdBr_2_.

### 2.4 Multiple‐Mode Color‐Tunable Smart Persistent Luminescence

Synergistic effects of the multiple energy levels and diverse ultralong‐lived photoemission states based on both TADF and RTP endow the Cy‐CdCl_2_ with the multiple‐mode tunable persistent luminescence in response to temperature and excitation stimuli, enabling great opportunities for smart optical materials. The temperature‐dependent phosphorescence mentioned above, when induced in Cy‐CdCl_2_ by 365 nm excitation, produces a main emission peak which is red‐shifted from 488 to 513 nm. The color of the afterglow changes from cyan to green as the temperature increases from 97 to 377 K (Figure 3d). The long‐lived persistent luminescence is observable even several seconds after excitation from the 365 nm UV lamp has been switched off. When *λ*_ex_ is adjusted to 395 nm, the main emission peak of the halide material is blue‐shifted from 510 to 435 nm. The color of the afterglow changes from green to sky blue as the temperatures increases from 97 to 377 K. The CIE coordinates of the afterglows are shown in Figure 4c and indicate colors from green to blue, which are in good agreement with naked‐eye observations.

Interestingly, the afterglow of the organic‐metal halide material is observed to evolve with time. At longer times after halting irradiation, the afterglow colors of Cy‐CdCl_2_ solids progressively vary from green to cyan and then to blue (Figure S17, Supporting Information). To acquire deeper insights, we further mapped the time‐resolved afterglow emission spectra of Cy‐CdCl_2_. As illustrated in Figure S18 (Supporting Information), with prolonged time, blue regions grow in relative intensity while green afterglow intensities are gradually weakened. To be precise, when *t*_off_ is 0 s, the delayed emission maximum is around 513 nm, reflecting the persistent RTP from the enol‐form of cytosine with a long lifetime of 125.7 ms (Figures 2c and 4e). When *t*_off_ is 2 s, the emission from the enol form decreases substantially, leading to a relative balance of green and blue light emissions centered at 513 nm, which manifests as a mixed‐color cyan afterglow. When the *t*_off_ is 2.5 s, the blue TADF at 435 nm becomes predominant, rendering the afterglow color blue.

### 2.5 Information Encryption and Logic Operation Applications

Exploiting the tunable multicolor emission and excitation‐/time‐dependent afterglows may provide a promising means by which these halide materials can serve for advanced data encryption and anti‐counterfeiting applications. Impressively, 2D Cy‐CdCl_2_ microcrystals show novel excitation‐related and space/time‐resolved properties together with good luminescence visibility, which combine the advantages of traditionally individual space‐resolution (e.g., 1D fluorescent micro/nanostructures) and time‐resolution (e.g., powders with long‐life emission) at the single crystal level.^[^ ^61^, ^62^, ^63^ ^]^

**Figure 7:**
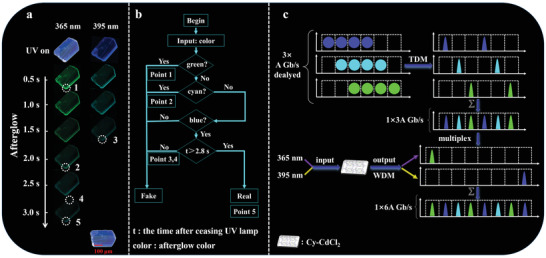
Schematic diagram of the information encryption and decryption processes. a) Long‐persistent emission images of microcrystal Cy‐CdCl_2_ under various excitation wavelengths. b) Illustration of operation of the optical logic gates whose operation is based on the dual‐resolved properties of the organic‐metal halide materials (Cy‐CdCl_2_ microcrystal). c) Scheme of time‐ and wavelength‐division multiplexing (TDM and WDM) models applicable for optical communications, made possible through a 2D Cy‐CdCl_2_ microsheet.

Firstly, the modeling protocol for predicting the crystal morphology of the Cy‐CdCl_2_ is enabled by the equilibrium morphology method (Figure S19, Supporting Information). The simulated crystal morphology provides four fast growing crystal facets of {1 0 1}, {0 1 2}, {1 1 0}, and {0 1 0} with the surface areas of 30.24%, 11.36%, 4.39%, and 3.95%, respectively (Table S10, Supporting Information). The simulated aspect ratio of the Cy‐CdCl_2_ crystal is calculated to be 7.875, corresponding approximately to experimental observations by luminescent microscopy (**Figure** **7**a). The Cy‐CdCl_2_ microsheets maintain the visible afterglow emission of different colors throughout a timescale of at least 3 s for the time‐resolved characteristics.

Having examined the time‐resolved characteristics of the Cy‐CdCl_2_ micro/nanosheets, we set out to study the space‐resolved characteristics, which manifest as 2D persistently luminescent waveguide characteristics. As shown in Figure 6a, a single Cy‐CdCl_2_ microcrystal shows multicolor afterglow turning from bright green to greenish‐cyan and to blue at the edge of the microsheet under 365 nm UV irradiation. To the best of our knowledge, while typical 1D fluorescent optical waveguides formed from pure organic crystals or inorganic–organic hybrid materials are well‐developed,^[^ ^64^ ^]^ active 2D optical waveguide materials are still quite limited, especially those capable of the time‐resolved triple color conversion based on the time‐dependent afterglow. As a result of the combined space/time‐resolved characteristics, the luminescence signal of the 2D crystal is observed to decrease gradually from the center to the edge of the microsheet following the switching off of UV irradiation at different wavelengths. More importantly, during this period, the edges of microsheets show polychromatic afterglow colors at different excitation wavelengths and times. Therefore, we have exploited this phenomenon to design an optical logic gate on a Cy‐CdCl_2_ crystal as a microchip (Figure 7b). The afterglow colors on the edge of the microchip were recorded for 0–3 s after turning off UV irradiation of different wavelengths. We set the color as the input value. Firstly, an input of green produces either a “YES,” which is a fake message (point 1), or a “NO,” at which point cyan is input; if the response is “YES,” it is a fake message (point 2). Continued input of blue that produces “NO” indicates a fake message, while for “YES,” a further input for t > 2.8 s could produce “NO,” which is a fake message (points 3 and 4), or “YES,” which is the last option for real information (point 5). In this manner, the space/time‐resolved characteristics demanded by multi‐anti‐counterfeiting technology can be obtained within the 2D metal halide hybrid at the microcrystal level.

By further borrowing the strategies of the established time‐ and wavelength‐division multiplexing (TDM and WDM) models in optical communications, the unique time‐ and excitation‐dependent persistent luminescence for the 2D Cy‐CdCl_2_ microsheet can serve as a high‐density information storage medium. To be precise, the initial information transmission rate of *A* Gb/s for the optical waveguide can be enhanced to 3A Gb/s due to the time‐dependent evolution of blue/green/cyan triple‐color. Exploiting two excitation inputs (365 and 395 nm) can further lead to 6A Gb/s. In principle, given divisible *N* excitation resources and wide‐range *M* channel wavelengths, the Cy‐CdCl_2_ could achieve even higher transmission capacity and speeds (i.e., more than *NMA* Gb/s), which could be enhanced further if combined with 2D polarization or spatial‐division multiplexing (Figure 7c). Moreover, the materials and methods demonstrated in this work might serve to improve the technology which is currently most promising namely, time‐wavelength interleaving in which the optical signals at different time delays are based on chromatic dispersion generated by optical frequency combs.^[^ ^65^, ^66^ ^]^ The excitation‐ and time‐dependent colorful emissive information here may enable a more secure approach as a result of the intrinsically photophysical properties of Cy‐CdCl_2_. Consequently, these metal halide micro/nanosheets with space/time dual‐resolved persistent RTP properties have great potential to serve as a new tool for luminescent visualization affording processing of in situ optical logic operations and high‐dimensional information storage.

## 3 Conclusion

In summary, we have developed a facile strategy to obtain colorful long‐afterglow materials with both forward (prompt mode) and reversed (delayed mode) excitation dependent luminescence, benefitting from the efficient ESIPT process within a series of Zn/Cd cytosine halides (Figures S20–S24, Supporting Information). The presence of metal and halides simultaneously promotes and tunes the SOC and the ISC process, and the strong coordination bonds further inhibit non‐radiative relaxation to promote triplet emission. Moreover, the time‐dependent triple‐chromatic afterglow conversion can be adjusted by controlling the balance of the enol to keto transition during the delayed photoemission process. Owing to the multi‐mode responsive (excitation‐, temperature‐, and time‐dependent) afterglow, the organic‐metal halides can serve as new types of smart multi‐responsive persistent luminescence materials. Furthermore, by manipulating the space/time dual‐resolved RTP and TADF emission within the 2D microsheets, the metal halide crystals can also be used for information encryption and optical logic gate. These findings can inspire future efforts to rationally design and fabricate smart and processable crystalline materials with colorful, excitation wavelength‐, temperature‐, and time‐dependent afterglow suitable for diverse advanced photofunctional applications.

## 4 Experimental Section

### Materials

All chemicals were reagent grade and used as purchased without further purification. pure CdCl_2_, ZnCl_2_, CdBr_2_, ZnBr_2_, 2‐methoxy‐4‐pyrimidinamine and cytosine were purchased from Sigma Chemistry Co. Ltd. and used without further purification.

### Synthesis of Metal Cytosine Halides

Reaction of CdCl_2_ċ2.5H_2_O (57 mg, 0.25 mmol) and cytosine (55 mg, 0.5 mmol) in water solution (10 mL) was carried out to afford bulk block crystals (named as Cy‐CdCl_2_) with the yield of 86% (based on Cd). Substituting ZnCl_2_ (34 mg, 0.25 mmol), ZnBr_2_ (57 mg, 0.25 mmol), and CdBr_2_ċ4H_2_O (86 mg, 0.25 mmol) metal salts for CdCl_2_ under similar conditions led to the formation of Cy‐ZnCl_2_ with the yield of 74% (based on Zn), Cy‐ZnBr_2_ with the yield of 69% (based on Zn) and Cy‐CdBr_2_ with the yield of 58% (based on Cd) respectively. Reaction of ZnCl_2_ (54 mg, 0.4 mmol) and 2‐methoxy‐4‐pyrimidinamine (50 mg, 0.4 mmol) in water solution (10 mL) was carried out to afford bulk block crystals (named as Me‐Cy‐ZnCl_2_) with the yield of 62% (based on Zn).

### Characterization

FT‐IR spectra were recorded in the range of 4000−400 cm^−1^ on a Shimadzu IRAffinity‐1. UV–vis absorption spectra were obtained on a Shimadzu UV‐3600 spectrophotometer at room temperature. Data were collected in the wavelength range of 200–800 nm. Photographs for the four hybrid materials were taken under OLYMPUS IXTI fluorescence microscope. All the relevant PL tests and time‐resolved lifetime were conducted on an Edinburgh FLS 980 fluorescence spectrometer.

### X‐Ray Crystallography

The single‐crystal X‐ray diffraction data of Cy‐ZnBr_2_ and Me‐Cy‐ZnCl_2_ were collected on a Rigaku XtalLAB Synergy diffractometer at 100(10) K with Cu‐K*α* radiation (*λ* = 1.54184 Å). SHELX‐2016 software was used to solve and refine the structure.^[^ ^67^ ^]^ Crystallographic data for Cy‐ZnBr_2_ and Me‐Cy‐ZnCl_2_ are listed in Table S1 (Supporting Information). Selected bond lengths and angles are listed in Tables S2 and S3 (Supporting Information). Full crystallographic data for Cy‐ZnBr_2_ and Me‐Cy‐ZnCl_2_ have been deposited with the CCDC (2117632, 2117633). The crystal structure data of Cy‐CdCl_2_ (1230021),^[^ ^68^ ^]^ Cy‐CdBr_2_ (177174),^[^ ^69^ ^]^ and Cy‐ZnCl_2_ (1134846)^[^ ^70^ ^]^ were downloaded from the Cambridge Crystallographic Data Centre.

### Calculation Details

Computational studies were performed using the Materials Studio.^[^ ^71^ ^]^ For the crystal morphology prediction, the vacuum morphology was executed using the Morphology module. The Bravais–Friedel–Donnay–Harker (BFDH) method^[^ ^72^ ^]^ was carried out and a face list was created using morphology simulation, which gave *hkl* values of all important faces with their respective d_hkl_. The molecular orbital calculations were performed with the periodic density functional theory (DFT) method using the Dmol3^[^ ^73^ ^]^ module. The initial configuration was fully optimized by the Perdew–Wang (PW91)^[^ ^74^ ^]^ generalized gradient approximation method with the double numerical basis sets plus polarization function (DNP). The core electrons for metals were treated by effective core potentials. The self‐consistent field converged criterion was within 1.0 × 10^−5^ hartree atom^−1^ and the converging criterion of the structure optimization was 1.0 × 10^−3^ hartree bohr^−1^. The Brillouin zone was sampled by 1 × 1 × 1 k‐points.

## Conflict of Interest

The authors declare no conflict of interest.

## Supporting information

Supporting Information

## Data Availability

The data that support the findings of this study are available from the corresponding author upon reasonable request.
