# Supplementary material for: Multi‐Mode and Dynamic Persistent Luminescence from Metal Cytosine Halides through Balancing Excited‐State Proton Transfer
Source: Adv Sci (Weinh). 2022 Apr 10;9(16):2200992. doi: 10.1002/advs.202200992 (PMC9165479; doi:10.1002/advs.202200992)
Supplement: Supplementary file 1 — Supporting Information [file ADVS-9-2200992-s001.pdf]

ADVANCED SCIENCE
Open Access

Supporting Information

for *Adv. Sci.*, DOI 10.1002/advs.202200992

Multi-Mode and Dynamic Persistent Luminescence from Metal Cytosine Halides through Balancing Excited-State Proton Transfer

*Guowei Xiao, Xiaoyu Fang, Yu-Juan Ma and Dongpeng Yan**

Supporting Information

Multi-mode and Dynamic Persistent Luminescence from Metal Cytosine Halides through Balancing Excited-State Proton Transfer

*Guowei Xiao, Xiaoyu Fang, Yu-Juan Ma, Dongpeng Yan**

Beijing Key Laboratory of Energy Conversion and Storage Materials, College of Chemistry, and Key Laboratory of Radiopharmaceuticals, Ministry of Education, Beijing Normal University, Beijing 100875, People's Republic of China

E-mail: yandp@bnu.edu.cn

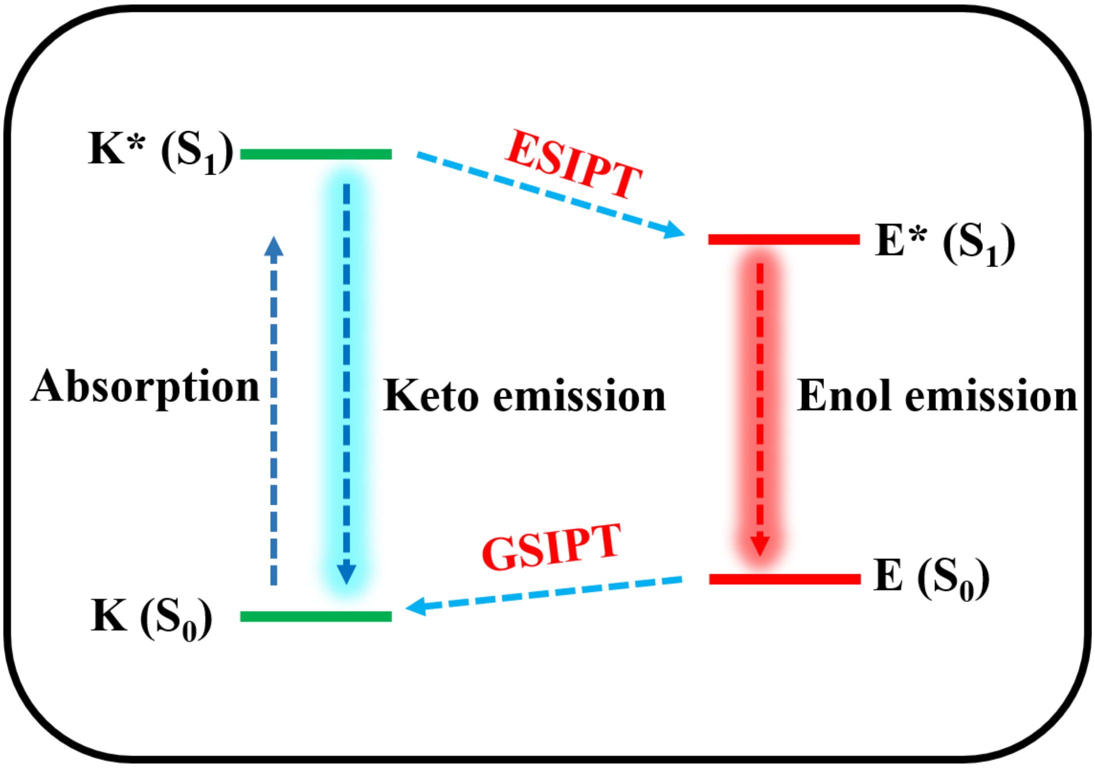

Figure S1. Schematic representation of classical ESIPT photocycle.

The classical diagram of the ESIPT involves the conventional four-level state energy cycle: fast proton transfer reaction from the proton donor to the proton acceptor takes place along the excited-state potential energy surface via the intramolecular H-bond, leading to a tautomeric transformation from the excited keto form (K∗) to the excited enol form (E∗). After decaying radiatively to the ground state, reverse proton transfer occurs to their initial K form. Thus, typically, the ESIPT process undergoes a red-shift of the photoemission compared with the keto form.

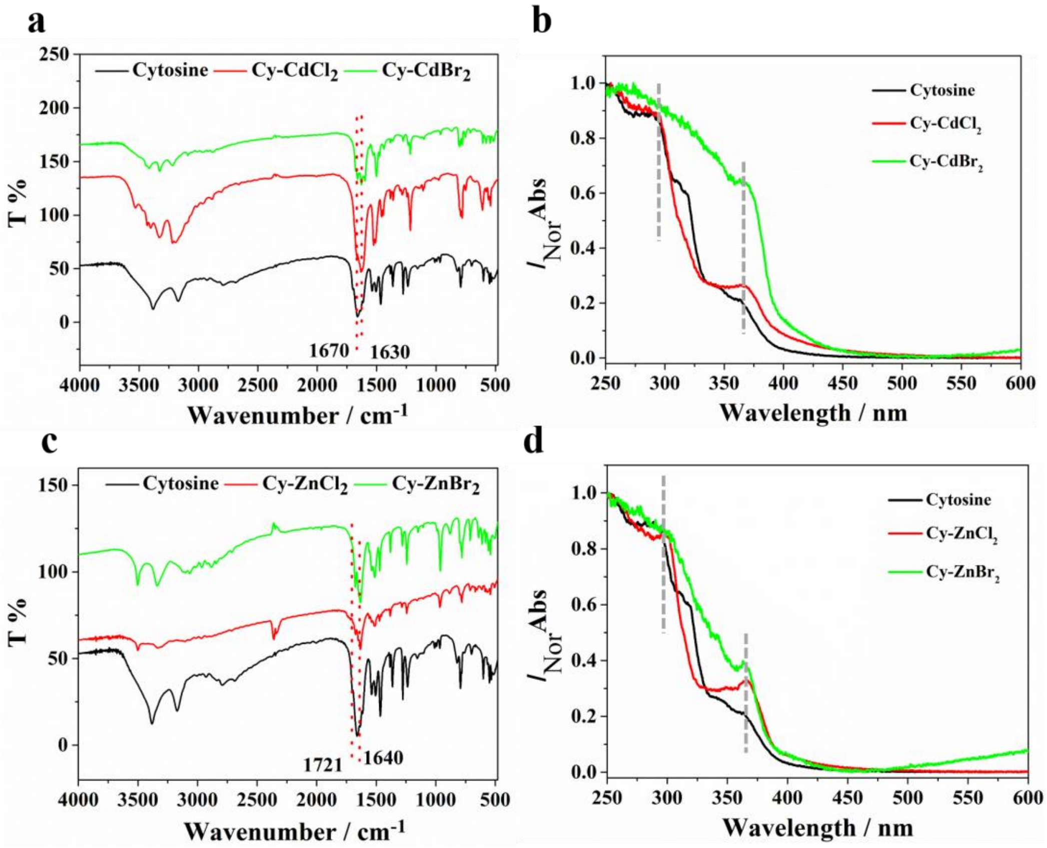

Figure S2. (a, c) IR plots and (b, d) absorption spectra of **Cytosine**, **Cy-CdCl$_2$**, **Cy-CdBr$_2$**, **Cy-ZnCl$_2$** and **Cy-ZnBr$_2$**.

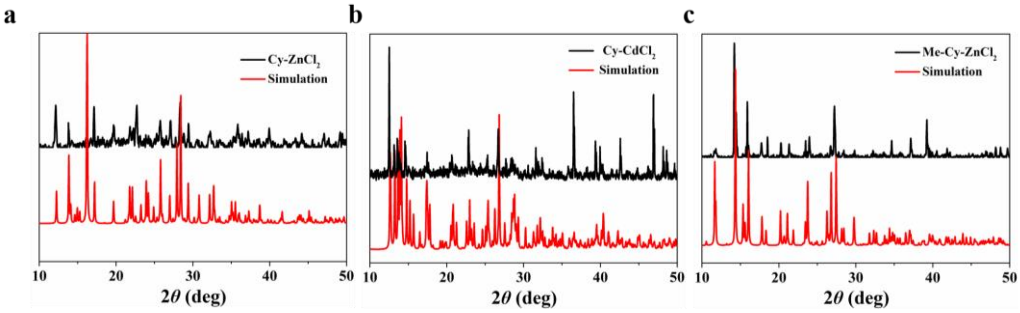

Figure S3. The simulated (red) and as-synthesized (black) PXRD patterns for **Cy-ZnCl$_2$, Cy-CdCl$_2$** and **Me-Cy-ZnCl$_2$**.

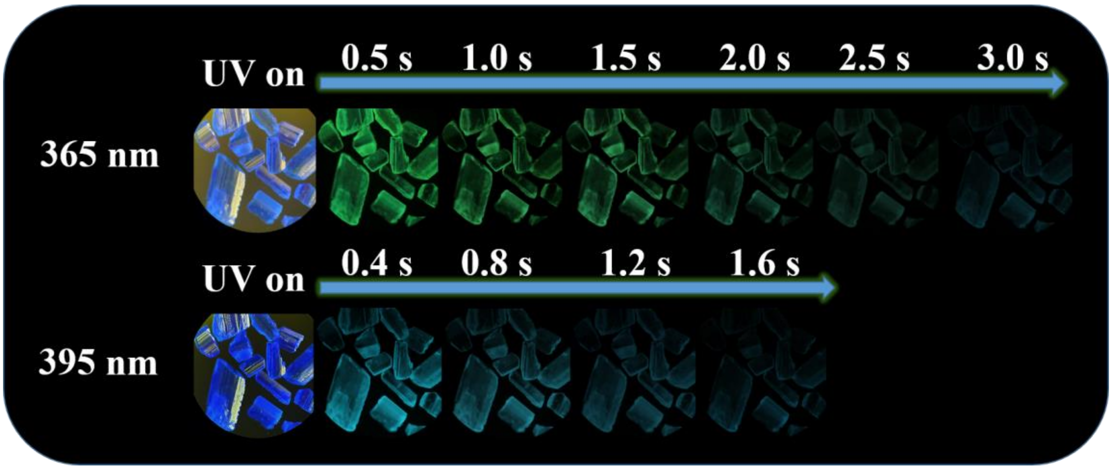

Figure S4. Afterglow photographs of **Cy-CdCl$_2$** crystals under different UV excitation wavelengths.

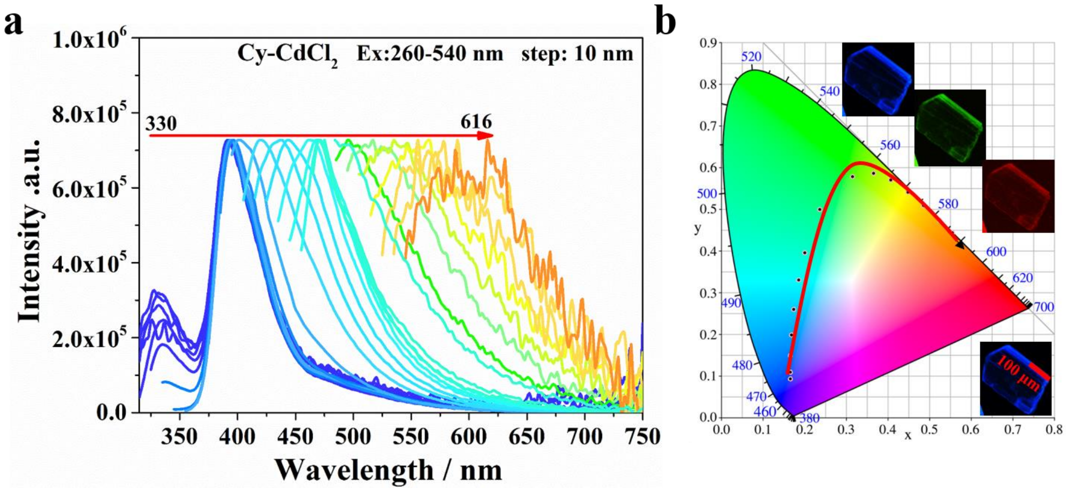

Figure S5. (a) Prompt PL emission spectra and (b) CIE coordinate of **Cy-CdCl$_2$** with different excitation wavelengths. Insert: The fluorescent photographs of **Cy-CdCl$_2$** (λ_{ex} = 395, 435, and 500 nm).

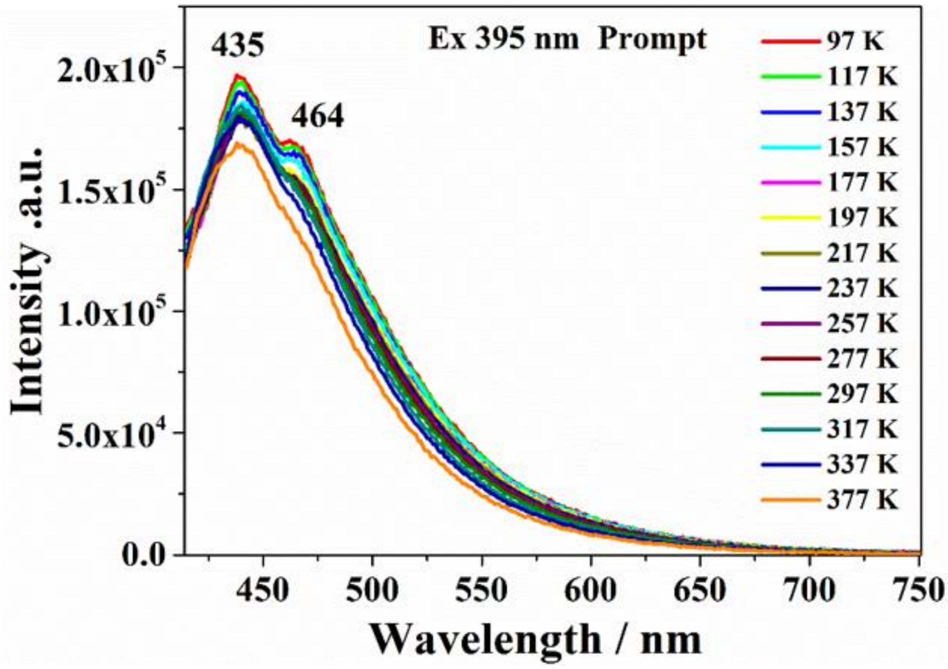

Figure S6. The PL spectra of **Cy-CdCl$_2$** at different temperatures ranging from 97 K to 377 K under the prompt mode with 365 nm excitation wavelength.

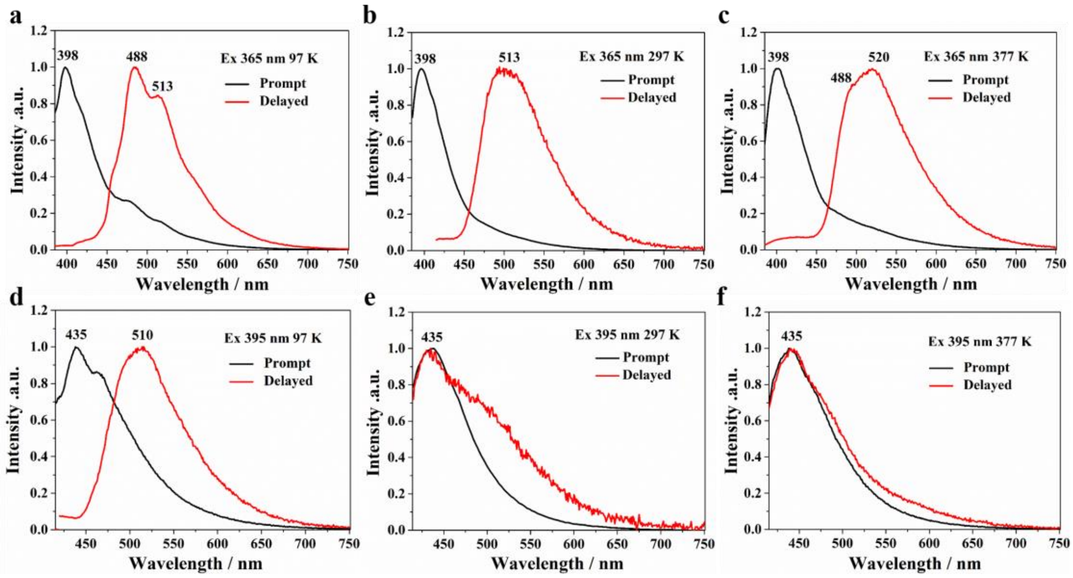

Figure S7. (a-c) Prompt and delayed emission spectra of **Cy-CdCl$_2$** at different temperature under 365 nm UV excitation and (d-f) 395 nm UV excitation.

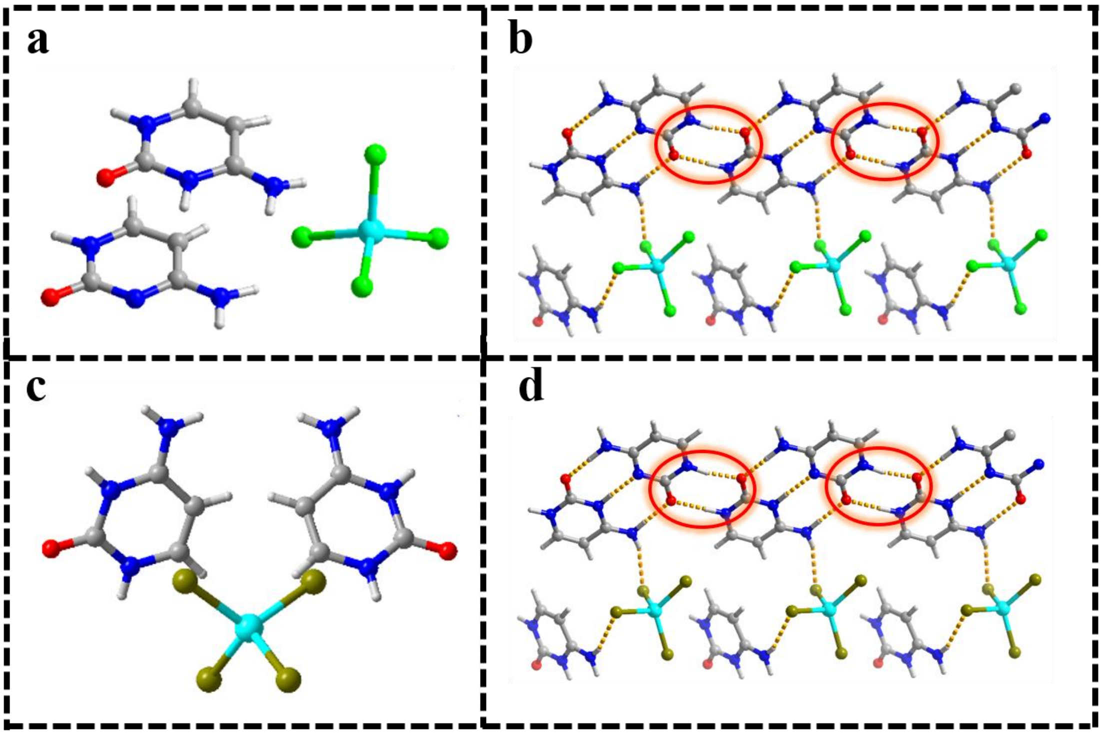

Figure S8. (a) The asymmetric unit of **Cy-ZnCl$_2$** and (c) **Cy-ZnBr$_2$**; The packing mode of (b) **Cy-ZnCl$_2$** and (d) **Cy-ZnBr$_2$** with H-bonds and N-H$\cdots$X (X = Cl, Br) shown as dashed lines. The detailed distances of intermolecular interactions were shown in Table S4, S8 and S9. (Teal: Cd; Green: Cl; Dark yellow: Br; Red: O; Blue: N; Gray-40%: C; Gray-20%: H)

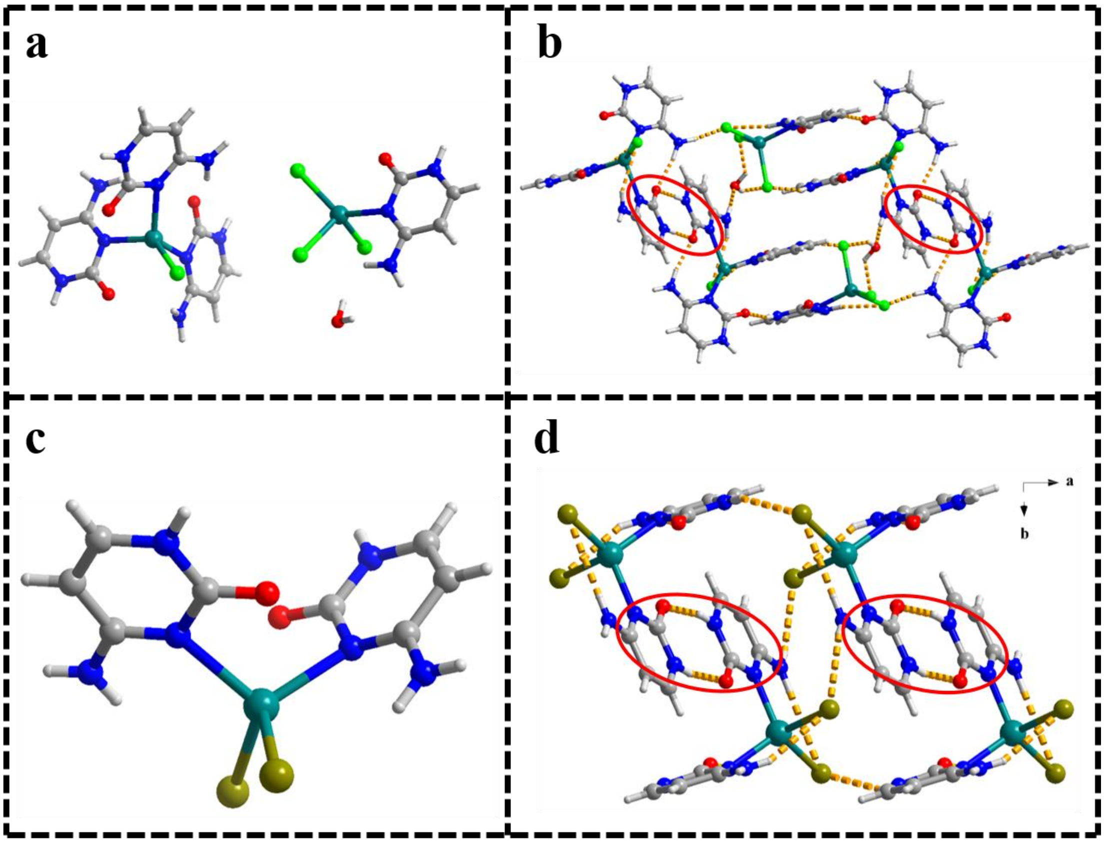

Figure S9. (a) The asymmetric unit of **Cy-CdCl₂** and (c) **Cy-CdBr₂**; The packing mode of (b) **Cy-CdCl₂** and (d) **Cy-CdBr₂** with H-bonds and N-H···X (X = Cl, Br) shown as dashed lines. The detailed distances of intermolecular interactions were shown in Table S6 and S7. (Teal: Cd; Green: Cl; Dark yellow: Br; Red: O; Blue: N; Gray-40%: C; Gray-20%: H)

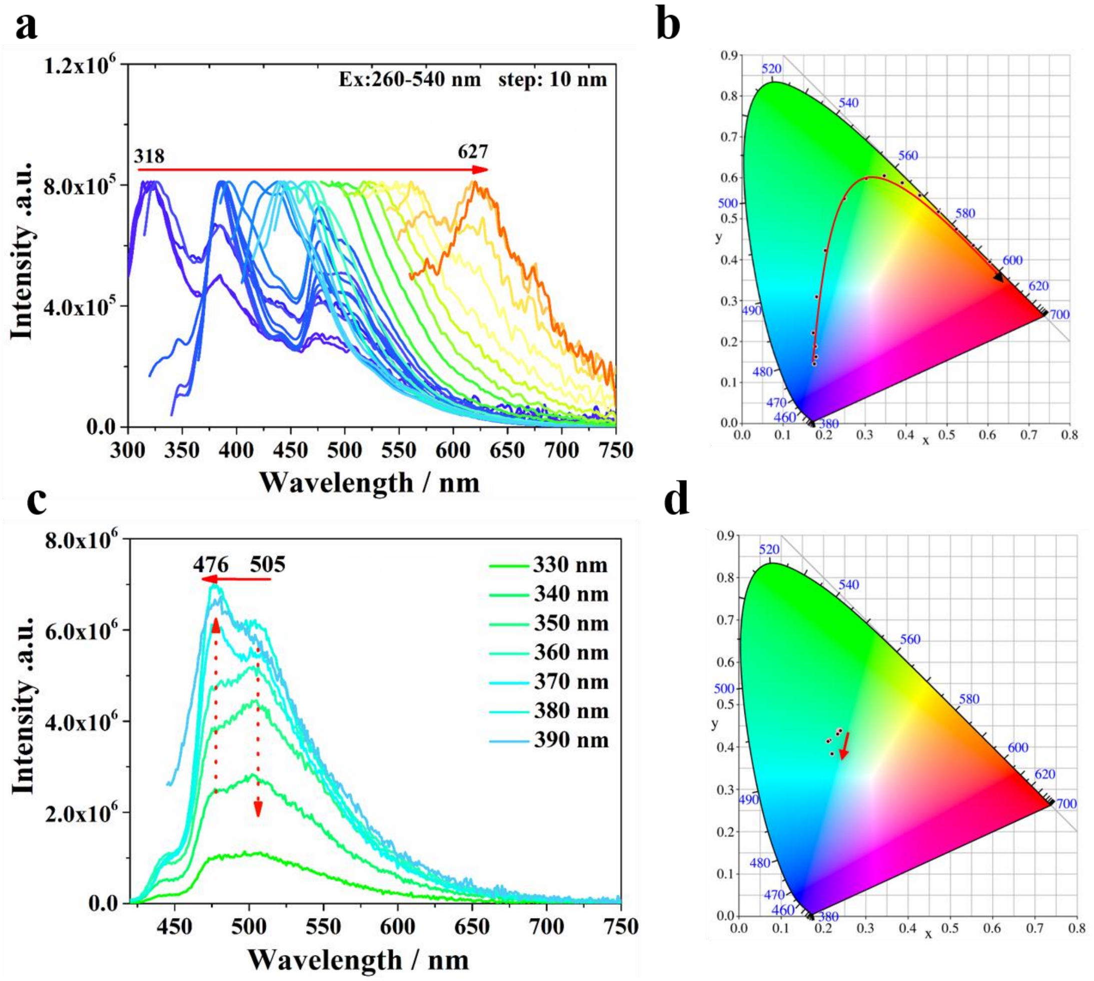

Figure S10. (a) Prompt PL emission spectra and (b) CIE coordinate of **Cy-ZnBr$_2$** with different excitation wavelengths; (c) Delayed PL emission spectra and (d) CIE coordinate of **Cy-ZnBr$_2$** with different excitation wavelengths.

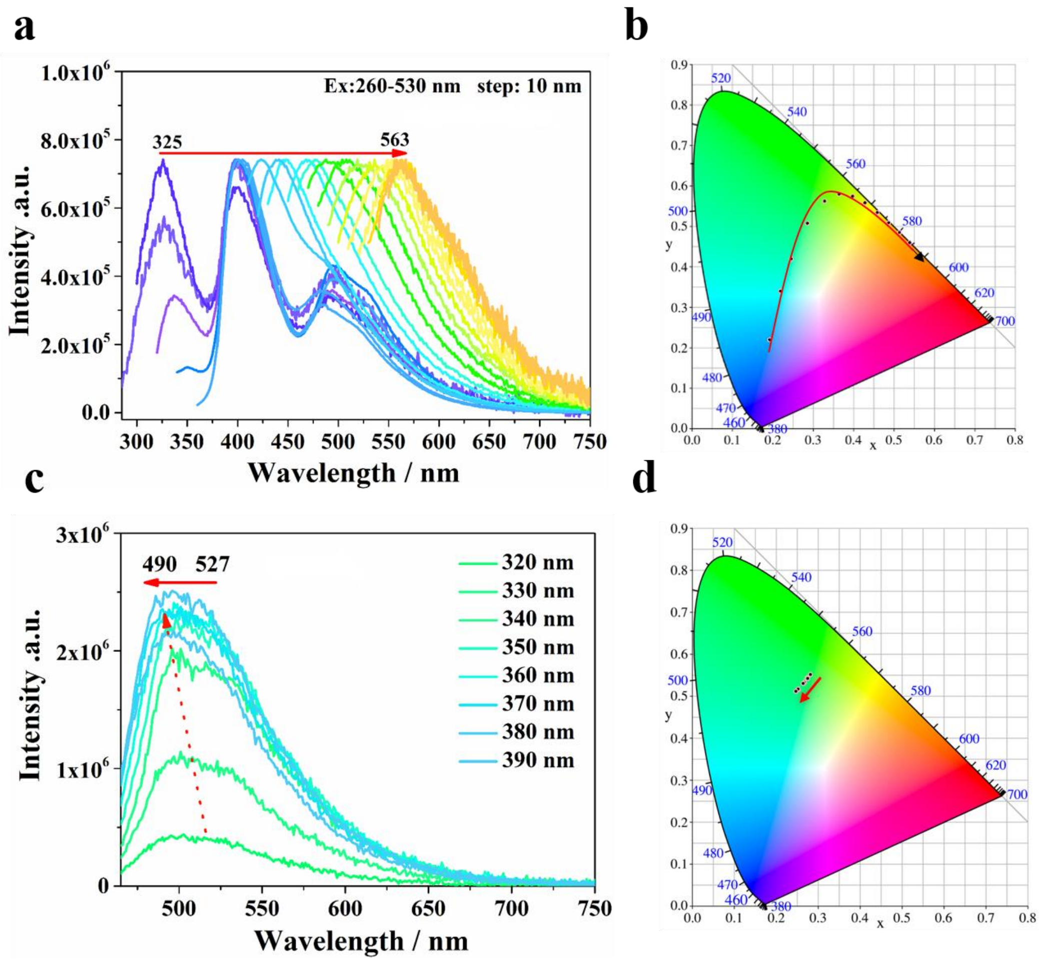

Figure S11. (a) Prompt PL emission spectra and (b) CIE coordinate of **Cy-CdBr$_2$** with different excitation wavelengths; (c) Delayed PL emission spectra and (d) CIE coordinate of **Cy-CdBr$_2$** with different excitation wavelengths.

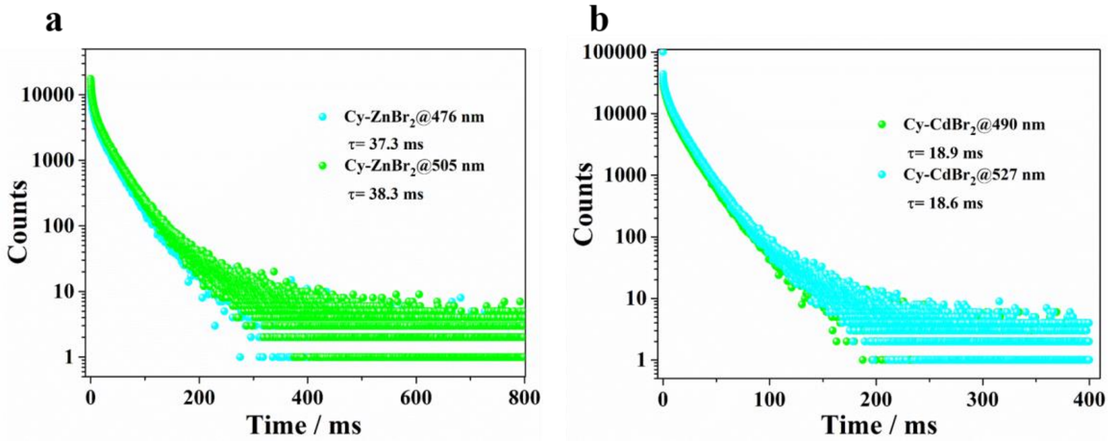

Figure S12. Decay curves obtained at room temperature for (a) **Cy-ZnBr$_2$** and (b) **Cy-CdBr$_2$**.

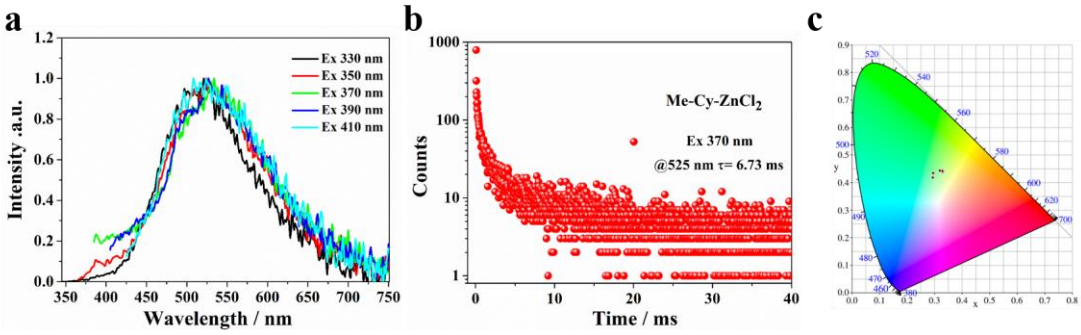

Figure S13. (a) Normalized delayed emission spectra of **Me-Cy-ZnCl$_2$** with different excitation wavelengths; (b) Decay curve of **Me-Cy-ZnCl$_2$** obtained at room temperature; (c) CIE coordinate diagram of **Me-Cy-ZnCl$_2$** by changing the excitation wavelengths.

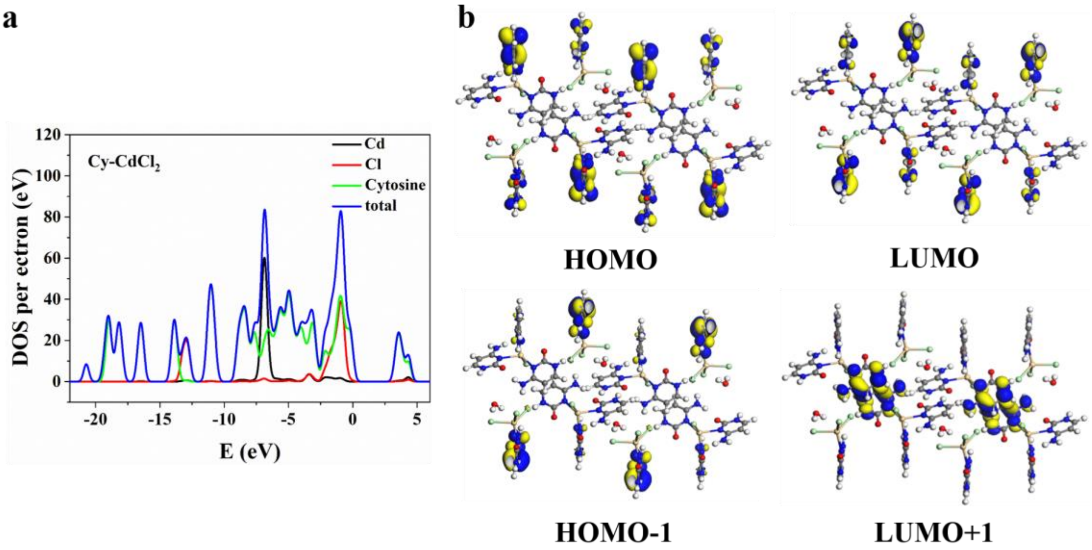

Figure S14. (a) Total/partial electronic density of states and (b) calculated molecular orbitals for **Cy-CdCl$_2$**.

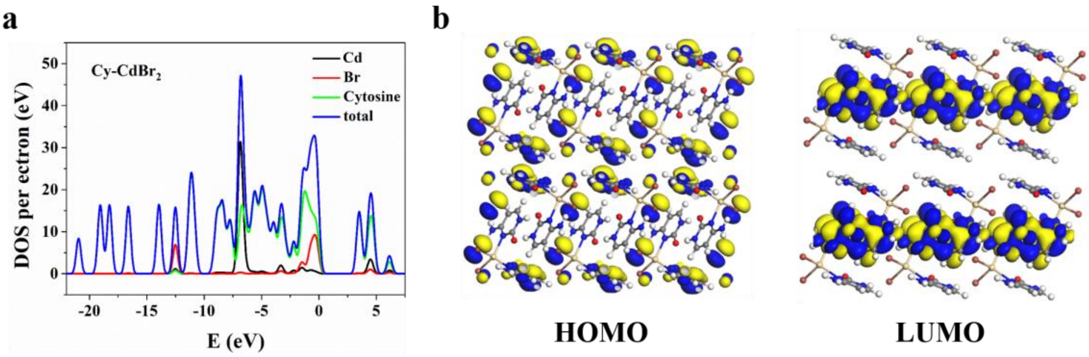

Figure S15. (a) Total/partial electronic density of states and (b) calculated molecular orbitals for **Cy-CdBr$_2$**.

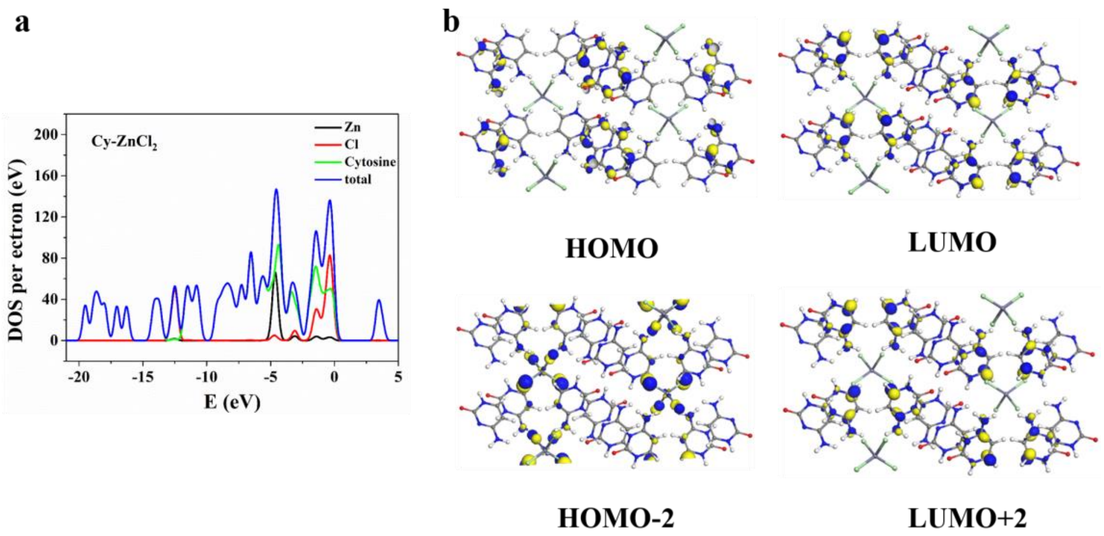

Figure S16. (a) Total/partial electronic density of states and (b) calculated molecular orbitals for **Cy-ZnCl$_2$**.

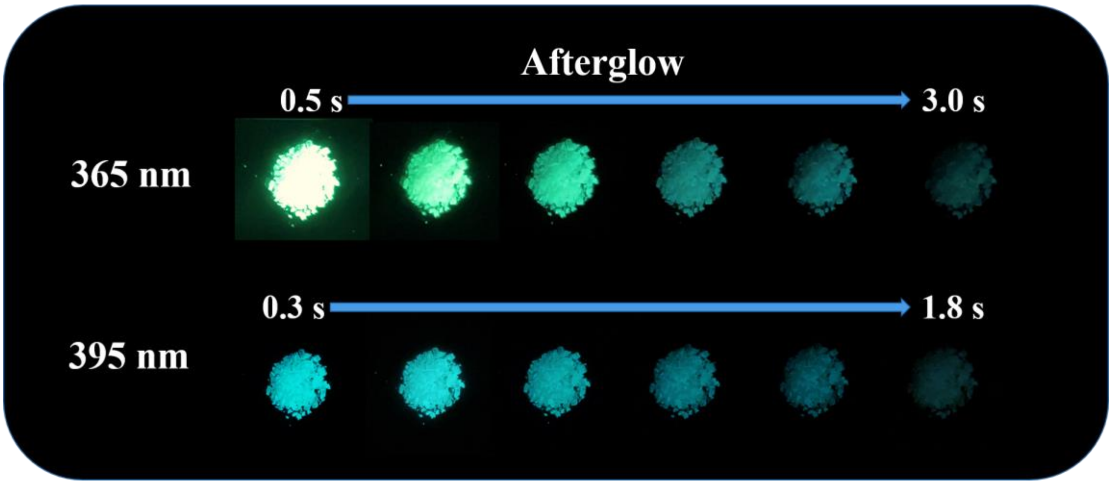

Figure S17. Afterglow photographs of **Cy-CdCl$_2$** powder under different UV excitation wavelengths.

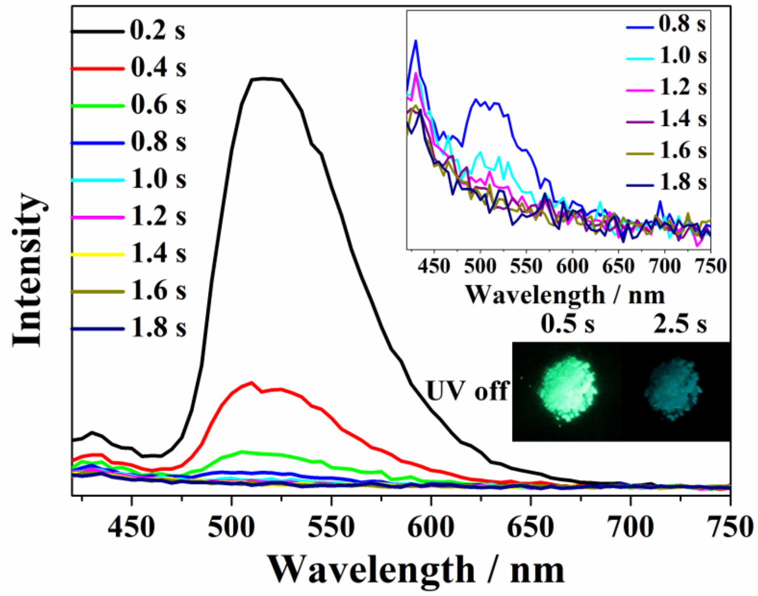

Figure S18. Time-dependent emission spectra of **Cy-CdCl$_2$** exported by time-resolved emission spectra (TRES). Insert: afterglow photographs captured after UV off for 0.5 s and 2.5 s.

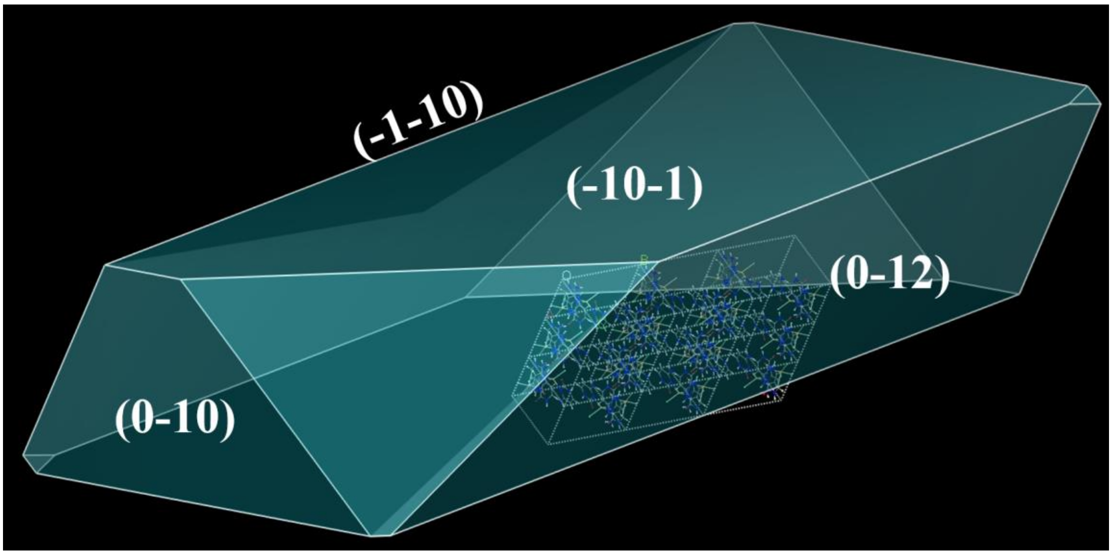

Figure S19. Predicted 2D crystal morphology and corresponding crystal faces for **Cy-CdCl$_2$** based on the equilibrium morphology method.

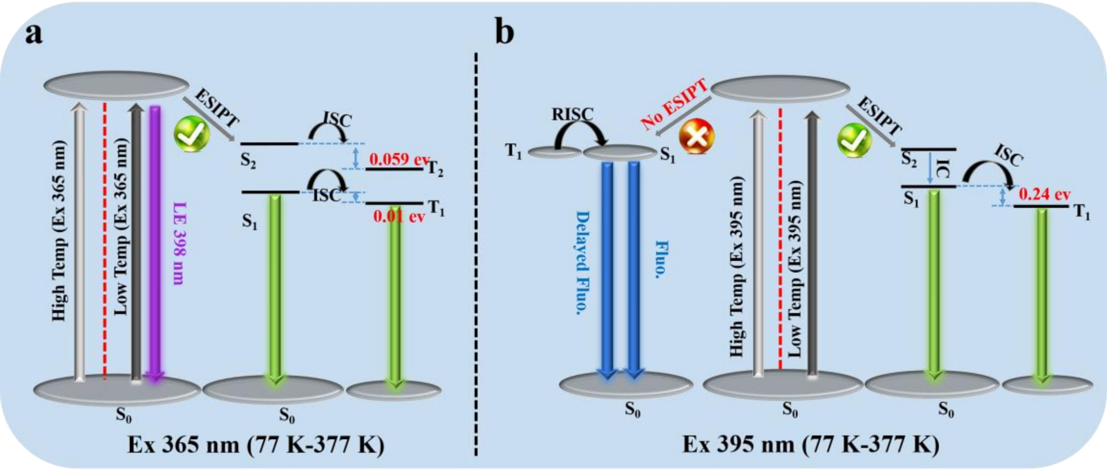

Figure S20. (a) Energy diagram of hybrid material **Cy-CdCl$_2$** for multicolor ultralong phosphorescence with the change of temperature ranging from 77 K to 377 K under 365 nm UV irradiation. (b) Energy diagram of hybrid material **Cy-CdCl$_2$** for multicolor ultralong phosphorescence with the change of temperature ranging from 77 K to 377 K under 395 nm UV irradiation.

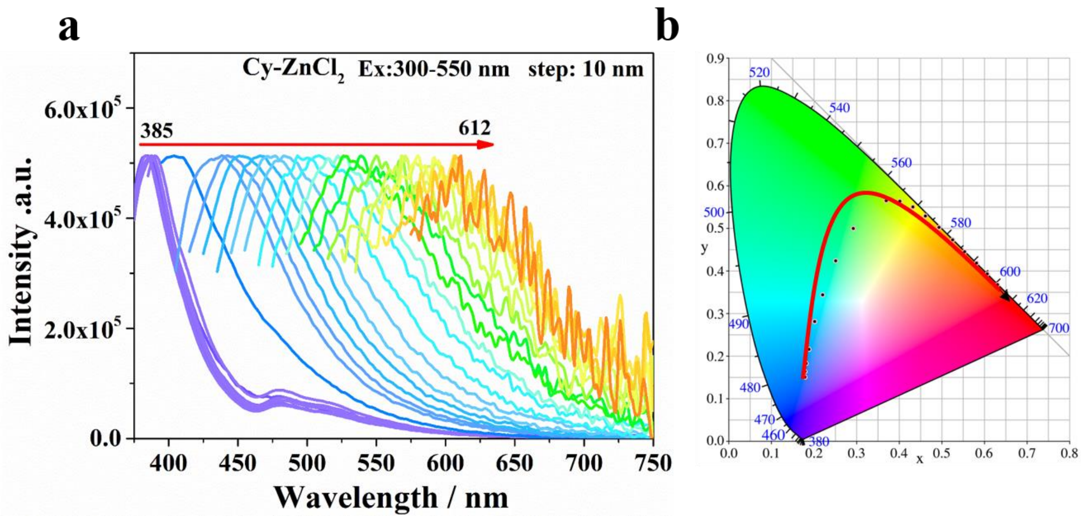

Figure S21. (a) Prompt PL emission spectra and (b) CIE coordinate of **Cy-ZnCl$_2$** with different excitation wavelengths.

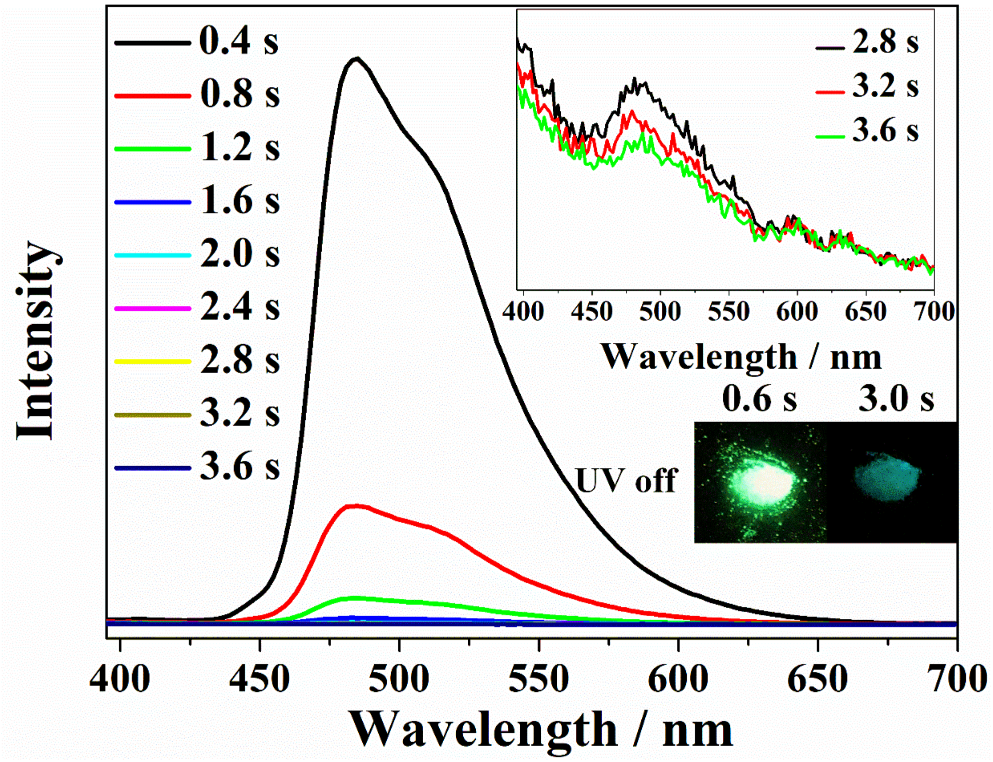

Figure S22. Time-dependent emission spectra of **Cy-ZnCl$_2$** exported by time-resolved emission spectra (TRES). Insert: afterglow photographs captured after UV off for 0.6 s and 3.0 s.

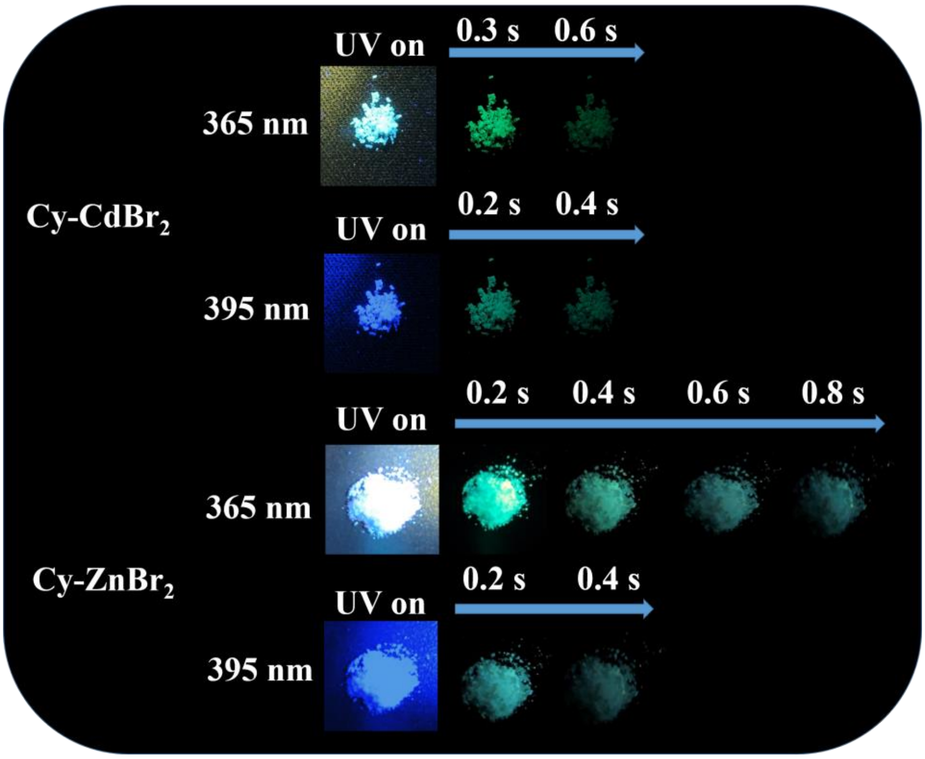

Figure S23. Afterglow photographs of **Cy-CdBr$_2$** and **Cy-ZnBr$_2$** under different UV excitation wavelengths.

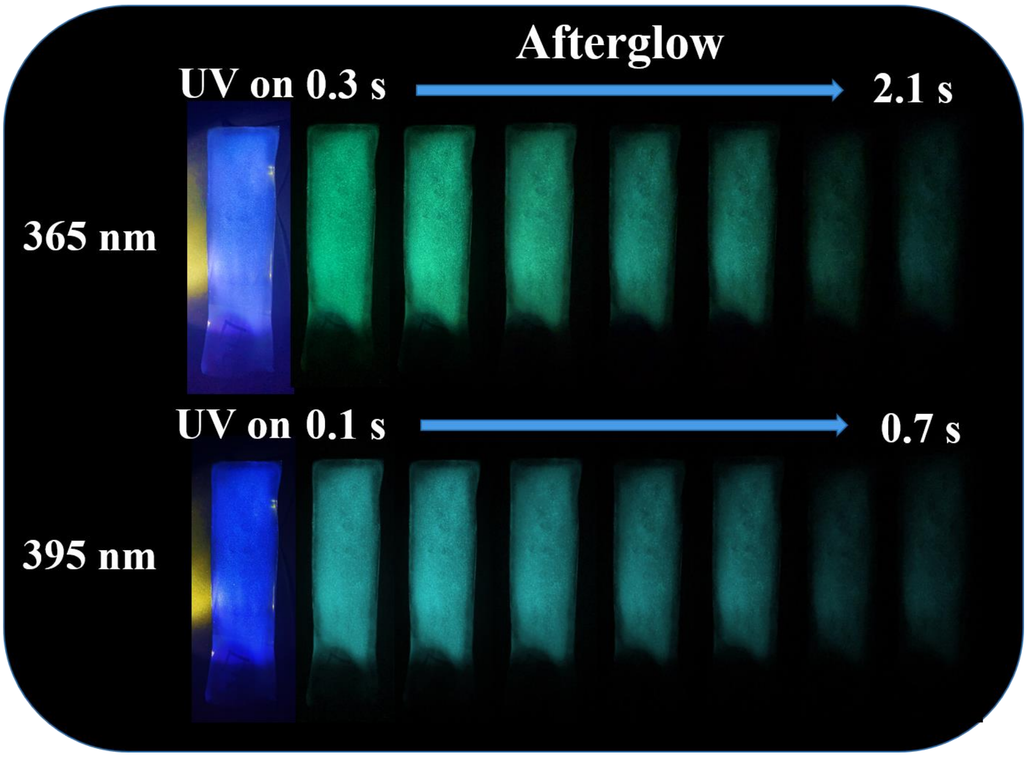

Figure S24. Afterglow photographs of **Cy-ZnCl$_2$** (20 mg) doping in styrene ethylene butylene styrene (SEBS, 60 mg) under different excitation wavelengths.

Table S1. The comparison of materials with the positively-forward excitation-dependent afterglow emission.

Reference Number	Sample Name	Tuning Range	Excitation Wavelength Range
	LIFM-WZ-7	(0.23, 0.24) to (0.48, 0.47)	310 to 440 nm
**1**	LIFM-WZ-8	(0.25, 0.26) to (0.49, 0.46)	310 to 440 nm
	LIFM-WZ-9	(0.28, 0.29) to (0.50, 0.48)	310 to 440 nm
	TMOT	452 to 505 nm	250 to 400 nm
**2**	DMOT	430 to 470 nm	250 to 400 nm
	CYAD	380 to 450 nm	250 to 390 nm
**3**	PVA-100-P4-1	468 to 522 nm	340 to 370 nm
**4**	SA-TPE	400 to 660 nm	254 to 365 nm
**5**	PCA-PVA	474 to 677 nm	380 to 349 nm
**6**	PVP-S	452 to 600 nm (77 K)	280 to 500 nm
**7**	PSSNa polymer	411 to 568 nm (77 K)	300 to 450 nm
**8**	a-CDs/BA	530 to 575 nm	350 to 500 nm

**9**	HA	452 to 539 nm	290 to 390 nm
**10**	Cd-TFTPA/NH_4F	417 to 533 nm	250 to 380 nm
	Cd/Mn-TFTPA/NH_4F	417 to 614 nm	250 to 380 nm
	Cd/Pb-TFTPA/NH_4F	435 to 550 nm	250 to 380 nm
**11**	MEL	461 to 494 nm	280 to 380 nm
**12**	$[Zn_3(D\text{-}Cam)_3(tib)_2]\cdot 2H_2O$	510 to 595 nm	290 to 430 nm
**This work**	Cy-$CdCl_2$	513 to 435 nm	365 to 395 nm
**This work**	Cy-$CdBr_2$	527 to 490 nm	320 to 390 nm
**This work**	Cy-$ZnCl_2$	513 to 428 nm	365 to 395 nm
**This work**	Cy-$ZnBr_2$	505 to 476 nm	330 to 390 nm

Table S2. Crystallographic data for **Cy-$ZnBr_2$** and **Me-Cy-$ZnCl_2$** at 100K.

	**Cy-$ZnBr_2$**	**Me-Cy-$ZnCl_2$**
Formula	$C_{16}H_{22}Br_4N_{12}O_4Zn$	$C_{10}H_{14}Cl_2N_6O_2Zn$
*M*r (g·mol^{-1})	831.46	386.54
Space group	*C2/c*	$P\bar{1}$
Crystal system	Monoclinic	Triclinic
a (Å)	7.09340(10)	7.8587(3)
b (Å)	15.1626(2)	8.7880(3)
c (Å)	24.4296(3)	11.6296(2)
α (°)	90	100.260(2)
β (°)	93.5180(10)	94.281(2)
γ (°)	90	103.589(3)
V (Å^3)	2622.56(6)	762.48(4)
Z	4	2
F(000)	1616	392
Dc (gcm^{-3})	2.106	1.684
μ (mm^{-1})	8.882	5.593
R_{int}	0.0282	0.0417
limiting indices	$-8\le h\le 8$, $-18\le k\le 18$, $-30\le l\le 30$	$-9\le h\le 9$, $-10\le k\le 11$, $-12\le l\le 14$
Collected reflections	19818	7931

Unique reflections	2578		3038	
GOF on F^2	1.046		1.058	
R_1, wR_2 [I>2$\sigma(I)$]	0.0196	0.0491	0.0336	0.0890
R_1, wR_2 [all data]	0.0201	0.0494	0.0349	0.0907

$^{a}R_1 = \sum ||F_o| - |F_c|| / \sum |F_o|$. ^{b} $wR_2 = \{\sum[w(F_o^2 - F_c^2)^2]/\sum w(F_o^2)^2\}^{1/2}$.

Table S3. Selected bond lengths (Å) and angles (°) for **Cy-ZnBr$_2$** at 100K.

**Cy-ZnBr$_2$**			
Br(2)-Zn(1)	2.4088(3)	Br(1)-Zn(1)	2.3886(3)
Br(1)#1-Zn(1)-Br(1)	117.12(2)	Br(1)#1-Zn(1)-Br(2)#1	107.814(8)
Br(1)#1-Zn(1)-Br(2)	107.274(8)	Br(1)-Zn(1)-Br(2)#1	107.275(8)
Br(1)-Zn(1)-Br(2)	107.814(8)	Br(2)-Zn(1)-Br(2)#1	109.395(19)

Symmetry codes: #1: -x+2, y, -z+1/2.

Table S4. Selected bond lengths (Å) and angles (°) for **Me-Cy-ZnCl$_2$** at 100K.

**Me-Cy-ZnCl$_2$**			
Zn(1)-N(4)	2.0213(17)	Zn(1)-Cl(2)	2.2677(5)
Zn(1)-N(1)	2.0267(17)	Zn(1)-Cl(1)	2.2722(5)
N(4)-Zn(1)-N(1)	111.74(7)	N(4)-Zn(1)-Cl(1)	110.04(5)
N(4)-Zn(1)-Cl(2)	108.99(5)	N(1)-Zn(1)-Cl(1)	108.05(5)
N(1)-Zn(1)-Cl(2)	107.22(5)	Cl(2)-Zn(1)-Cl(1)	110.77(2)

Table S5. Details of Hydrogen Bond Interactions in **Cy-ZnCl$_2$** at 295K.

D–H···A	d(D–H) (Å)	d(H···A) (Å)	d(D···A) (Å)	∠(DHA) (deg)
N(1)-H(1)...O(2)	0.99	1.84	2.8232(4)	172
N(2)-H(2)...N(5)	0.94	1.92	2.8618(4)	176
N(3)-H(3)...O(2)	0.92	1.87	2.7822(4)	178
N(3)-H(4)...Cl(2)	0.82	2.52	3.2995(5)	161
N(4)-H(7)...O(1)	1.01	1.79	2.7992(4)	170
N(6)-H(8)...O(1)	1.07	1.91	2.9553(4)	167

Table S6. Details of Hydrogen Bond Interactions in **Cy-ZnBr$_2$** at 100K.

D–H···A	*d*(D–H) (Å)	*d*(H···A) (Å)	*d*(D···A) (Å)	∠(DHA) (deg)
N(1)-H(1)...N(5)	0.86	2.01	2.862(2)	171
N(2)-H(2)...O(2)	0.86	1.98	2.830(2)	168
N(3)-H(3A)...O(2)	0.86	1.92	2.782(2)	176
N(3)-H(3B)...Br(2)	0.86	2.59	3.4325(18)	167
N(4)-H(4)...O(1)	0.86	1.92	2.780(2)	177
N(6)-H(6A)...O(1)	0.86	2.12	2.965(2)	168

Table S7. Details of Hydrogen Bond Interactions in **Cy-CdCl$_2$** at 295K.

D–H···A	*d*(D–H) (Å)	*d*(H···A) (Å)	*d*(D···A) (Å)	∠(DHA) (deg)
O(1)-H(1)...Cl(2)	0.96(2)	2.33(2)	3.227(4)	155(2)
O(1)-H(2)...Cl(1)	0.95(3)	2.51(3)	3.238(3)	134(2)
N(1)-H(3)...O(2)	0.963(17)	2.036(19)	2.953(3)	158(2)
N(1)-H(4)...Cl(3)	0.95(2)	2.65(2)	3.585(3)	169(3)
N(2)-H(5)...O(5)	0.954(18)	1.936(19)	2.847(3)	159(3)
N(2)-H(6)...Cl(3)	0.95(2)	2.33(3)	3.239(3)	161(3)
N(3)-H(7)...O(4)	0.95(3)	2.01(2)	2.884(4)	153(2)
N(3)-H(8)...Cl(4)	0.946(18)	2.76(3)	3.317(3)	119(3)
N(4)-H(9)...Cl(4)	0.956(15)	2.820(17)	3.705(3)	154(2)
N(4)-H(9)...N(3)	0.956(15)	2.60(3)	3.299(4)	130(2)
N(4)-H(10)...O(1)	0.95(2)	1.87(2)	2.815(4)	174(2)
N(5)-H(11)...O(3)	0.90	1.98	2.858(3)	167
N(7)-H(14)...Cl(1)	0.90	2.30	3.181(3)	169
N(9)-H(17)...Cl(2)	0.90	2.35	3.200(3)	159
N(11)-H(20)...O(5)	0.90	1.96	2.864(4)	177

Table S8. Details of Hydrogen Bond Interactions in **Cy-CdBr$_2$** at 100K.

D–H···A	*d*(D–H) (Å)	*d*(H···A) (Å)	*d*(D···A) (Å)	∠(DHA) (deg)
N(1)-H(1)...O(2)	0.86	1.91	2.758(8)	168
N(1')-H(1')...Br(3)	0.86	2.68	3.488(7)	157
N(4')-H(41')...Br(2)	0.86	2.90	3.699(8)	156
N(4')-H(42')...O(2')	0.86	2.15	2.983(9)	162

Table S9. Details of Hydrogen Bond Interactions in **Me-Cy-ZnCl$_2$** at 100K.

D–H···A	*d*(D–H) (Å)	*d*(H···A) (Å)	*d*(D···A) (Å)	∠(DHA) (deg)
N(3)-H(3A)...N(2)	0.86	2.34	3.161(2)	159
N(3)-H(3B)...Cl(1)	0.86	2.49	3.3062(18)	160
N(6)-H(6A)...Cl(1)	0.86	2.73	3.5687(18)	167
N(6)-H(6B)...Cl(2)	0.86	2.54	3.3400(19)	156

Table S10. Surface area percentage of important facets of **Cy-CdCl$_2$** crystal by equilibrium morphology method.

hkl	***d$_{hkl}$***	**Distance**	**Total facet area%**
**{1 0 1}**	6.18	46.56	30.24
**{-1 0 -1}**	6.18	46.56	30.24
**{0 1 2}**	5.99	118.76	11.36
**{0 -1 -2}**	5.99	118.76	11.36
**{1 1 0}**	5.65	159.79	4.39
**{-1 -1 0}**	5.65	159.79	4.39
**{0 1 0}**	13.36	236. 68	3.95
**{0 -1 0}**	13.36	236. 68	3.95

Table S11. Phosphorescence lifetimes (τ) of Cy-CdCl$_2$@513 nm (Ex365 nm) and Cy-CdCl$_2$@435 nm (Ex395 nm).

Component	Wavelength (nm)	τ_1 (ms)	A_1 (%)	τ_2 (ms)	A_2 (%)	<τ> (ms)	χ^2
**Cy-CdCl$_2$ @513 nm**	513	78.4	55.36	184.3	44.64	125.7	1.036
**Cy-CdCl$_2$ @435 nm**	435	30.9	52.11	172.7	47.89	98.8	1.237

$<\tau>=\Sigma A_j\tau_j$, j=1,2,3...

Table S12. Long persistent luminescence lifetimes (τ) of **Cy-CdCl$_2$**@510 nm and 435 nm at different temperature upon 395 nm lamp.

Temperature	Wavelength (nm)	τ_1 (ms)	A_1 (%)	τ_2 (ms)	A_2 (%)	<τ> (ms)	χ^2
**257 K**	510	78.06	41.29	256.5	58.71	182.8	1.221
	435	84.24	55.63	310.6	44.37	184.7	1.300
**277 K**	510	63.83	48.71	226.9	51.29	147.6	1.205
	435	71.1	50.07	304.04	49.93	187.3	1.297
**297 K**	510	42.89	42.59	165.39	57.41	113.3	1.047
	435	44.84	37.82	208.75	62.18	146.8	1.257
**317 K**	510	86.74	100			86.74	1.218
	435	34.83	35.79	154.1	64.21	111.4	1.293
**337 K**	510						
	435	38.38	45.57	157.45	54.43	103.16	1.233
**377 K**	510						
	435	19.37	40.12	93.12	59.88	63.46	1.053

$\langle\tau\rangle = \sum A_j\tau_j$, j=1,2,3...

Table S13. Phosphorescence lifetimes (τ) of **Cy-CdCl$_2$** at 513 nm under 365 nm UV lamp and Fluorescence lifetime of **Cy-CdCl$_2$** at 435 nm under 395 nm UV lamp at different temperature.

	Temperature	Wavelength (nm)	τ_1 (ms)	A_1 (%)	τ_2 (ms)	A_2 (%)	<τ> (ms)	χ^2
	**97 K**	513	132.03	41.1	490.52	58.9	342.7	1.259
	**117 K**	513	107.43	54.55	291.78	45.45	191.2	1.245
	**137 K**	513	99.29	56.8	288.08	43.2	180.2	1.278
	**157 K**	513	96.97	58.84	279.82	41.16	172.2	1.234
	**177 K**	513	93.9	58.5	255.42	41.5	161	1.193
	**197 K**	513	80.18	56.67	238.96	43.33	149	1.298
Phosphorescence lifetimes	**217 K**	513	75.11	50.42	218.86	49.58	146.4	1.278
	**237 K**	513	86	62.19	228.55	37.81	139.9	1.075
	**257 K**	513	70.71	45.75	195.35	54.25	138.3	1.272
	**277 K**	513	69.06	44.05	184.09	55.95	133.3	1.268
	**297 K**	513	78.4	55.36	174.26	44.64	121.1	1.036
	**317 K**	513	71.45	57.88	122.86	42.12	93.2	1.017
	**337 K**	513	79	100			79	1.078

	**377 K**	513	63.5	100			63.5	1.095
	Temperature	Wavelength (nm)	τ_1 (ns)	A_1 (%)	τ_2 (ns)	A_2 (%)	$<\tau>$ (ns)	χ^2
Fluorescence lifetime	**297 K**	435	2.53	69.99	4.43	30.01	3	1.230

$<\tau>=\Sigma A_j\tau_j$, j=1,2,3...

References

[1] Z. Wang, C.-Y. Zhu, J.-T. Mo, X.-Y. Xu, J. Ruan, M. Pan, C.-Y. Su, *Angew. Chem. Int. Ed.* **2021**, *60*, 2526−2533.

[2] L. Gu, H. Shi, L. Bian, M. Gu, K. Ling, X. Wang, H. Ma, S. Cai, W. Ning, L. Fu, H. Wang, S. Wang, Y. Gao, W. Yao, F. Huo, Y. Tao, Z. An, X. Liu, W. Huang, *Nat. Photonics* **2019**, *13*, 406–411.

[3] Z. Wang, Y. Zhang, C. Wang, X. Zheng, Y. Zheng, L. Gao, C. Yang, Y. Li, L. Qu, Y. Zhao, *Adv. Mater*. **2020**, *32*, 1907355.

[4] X. Dou, T. Zhu, Z. Wang, W. Sun, Y. Lai, K. Sui, Y. Tan, Y. W. Zhang, Z. Yuan, *Adv. Mater*. **2020**, *32*, 2004768.

[5] Y. Su, Y. Zhang, Z. Wang, W. Gao, P. Jia, D. Zhang, C. Yang, Y. Li, Y. Zhao, *Angew. Chem. Int. Ed*. **2020**, *59*, 9967.

[6] H. Wang, H. Shi, W. Ye, X. Yao, Q. Wang, C. Dong, W. Jia, H. Ma, S. Cai, K. Huang, L. Fu, Y. Zhang, J. Zhi, L. Gu, Y. Zhao, Z. An, W. Huang, *Angew. Chem. Int. Ed*. **2019**, *58*, 18776–18782.

[7] S. Cai, H. Ma, H. Shi, H. Wang, X. Wang, L. Xiao, W. Ye, K. Huang, X. Cao, N. Gan, C. Ma, M. Gu, L. Song, H. Xu, Y. Tao, C. Zhang, W. Yao, Z. An, W. Huang, *Nat. Commun*. **2019**, *10*, 4247.

[8] W. Li, W. Zhou, Z. Zhou, H. Zhang, X. Zhang, J. Zhuang, Y. Liu, B. Lei, C. Hu, *Angew. Chem. Int. Ed*. **2019**, *58*, 7278–7283.

[9] Y. Wang, S. Tang, Y. Wen, S. Zheng, B. Yang, W. Z. Yuan, *Mater. Horiz.* **2020**, *7*, 2105.

[10] G. Xiao, B. Zhou, X. Fang, D. Yan, *Research* **2021**, *2021*, 9862327.

[11] Y. Miao, S. Liu, L. Ma, W. Yang, J. Li, J. Lv, *Anal. Chem.* **2021**, *93*, 4075–4083.

[12] Y.-J. Ma, X. Fang, G. Xiao, B. Lu, D. Yan, *Chem. Commun.* **2021**, *57*, 6684–6687.
